# Nudging to increase the uptake of cancer screening: scoping review of empirical studies

**DOI:** 10.1080/21642850.2026.2639784

**Published:** 2026-03-03

**Authors:** Līga Pūce, Vineta Silkāne

**Affiliations:** a Ventspils University of Applied Sciences, Ventspils, Latvia; b Vidzeme University of Applied Sciences, Vidzeme University of Applied Sciences Scientific Institute, Valmiera, Latvia

**Keywords:** Nudging, cancer screening, scoping review

## Abstract

**Background:**

Cancer screening is critical for early detection of cancer, yet achieving high participation rates remains a substantial challenge. This study aims to examine empirically tested nudging interventions and their effects on increasing cancer screening uptake.

**Method:**

A scoping review was conducted following PRISMA-ScR guidelines. Empirical studies on nudging to enhance cancer screening uptake, published from January 2008 to August 2025, were retrieved from Scopus, Web of Science, Science Direct, and EBSCO (including Academic Search Complete, Health Source: Nursing/Academic Edition, and MEDLINE) databases/search platforms. A complementary risk of bias analysis was performed for the selected studies.

**Results:**

Fifteen articles were included in this review. Most studies were carried out in real-life settings, with health system providers or professionals delivering nudges. The most common nudging techniques involved reminders combined with information provision. Results varied widely, ranging from no statistically significant effect to increases of several tens of percentage points, with a maximum increase of 65.1 percentage points. Randomised controlled trials with acceptable risk of bias demonstrated diverse outcomes, while other studies lacking a controlled approach reported positive results. Interventions that modified decision structures—such as altering options, adjusting consequences, or reducing effort—were the most effective and produced the largest increases. Intervention effects tended to be higher when resource-demanding components were included and/or healthcare professionals were involved.

**Conclusions:**

The review indicates that nudge interventions can positively influence cancer screening uptake. Further research is needed to explore various nudging strategies across diverse settings, particularly through rigorous randomised controlled trials.

## 1. Introduction

Cancer remains one of the leading causes of death globally, accounting for 9 to 10 million deaths annually—approximately one in six deaths worldwide (Ritchie & Mathieu, 2023; World Health Organization, 2024). In addition to its negative impact on population health, cancer contributes significantly to the global socioeconomic burden (Chen et al., 2023; Hofmarcher et al., 2020).

Screening programs are a critical component of cancer control, aimed at detecting precancerous changes or early-stage cancers in asymptomatic individuals. Early detection through screening can substantially reduce cancer-related morbidity and mortality (Lane & Smith, 2023; World Health Organization, 2022), thereby alleviating the broader socioeconomic burden associated with advanced disease. Despite the availability of organized screening programmes in many countries, participation rates often remain suboptimal (OECD, 2023), limiting the benefits of early cancer detection and treatment.

In the last decades, there has been a rising interest in applying knowledge gained from behavioural science and interventions, known as ‘nudges’, to shape health-related behaviours (Hanoch et al., 2017; Roberto & Kawachi, 2016; Thaler & Sunstein, 2008). A nudge is defined as *‘any aspect of the choice architecture that alters people’s behavior in a predictable way without forbidding any option or significantly changing their economic incentives’* (Thaler & Sunstein, 2008). Nudging aims to utilize insights from psychology of how people behave in decision situations, recognizing that cognitive biases and heuristics often lead to suboptimal decisions (Hansen, 2016; Tversky & Kahneman, 1974).

For example, when individuals face overwhelming options or complex information, people may resort to default choices or avoid making decisions altogether (Schwartz, 2004). By simplifying information and structuring choices, nudges can help to clarify the consequences of different options and thus facilitate better decision-making (Johnson et al., 2012). Moreover, nudging employs social norms and feedback mechanisms. When individuals are made aware of how their choices compare to those of others, or when they receive timely reminders about preferable actions, they are more likely to adjust their behaviours accordingly (Thaler & Sunstein, 2008). In this context, nudge interventions provide various strategies that modify the presentation of choices, making the desired options more salient and easier to select.

Nudge interventions include (1) changes in information provision, e.g. reframing, simplifying, providing feedback or social reference point, (2) changes in decision structure, e.g. changing choice defaults, option-related effort, composition of options or option consequences, and (3) decision assistance, e.g. providing reminders or encouraging self/public commitment (Münscher et al., 2016)—most of which can be utilised to target cancer screening uptake. See Appendix A for a detailed list of nudge intervention techniques.

Despite the growing application of behavioural insights in preventive healthcare and the existence of multiple empirical studies evaluating nudging strategies to improve cancer screening uptake, there remains a lack of synthesis and consensus on whether nudges are effective in increasing cancer screening uptake and under what circumstances. One literature review addressing nudging in the context of screening is that conducted by Hofmann and Stanak (2018); however, it focuses on the ethical considerations of nudging in health screening more broadly, without a specific emphasis on cancer or the effectiveness of particular interventions. Another recent review and meta-analysis specifically addressed digital interventions for the promotion of cancer screening behaviour and found a statistically significant positive effect—improved adherence to cancer screening behavior (OR = 1.81, 95% CI = 1.35–2.44, *p* < 0.001) (Wang et al., 2025). However, all included studies were conducted in the US, and the effectiveness of interventions outside the US and under conventional delivery conditions—such as mailing, calling, or direct communication—still remains insufficiently explored.

Meanwhile, reviews in related domains such as vaccination (Reñosa et al., 2021; Zhang & Jin, 2023), healthcare appointment adherence (Werner et al., 2023), and chronic disease management (Ahmed & McNamee, 2023; Kwan et al., 2020; Lee et al., 2024; Möllenkamp et al., 2019; Vande Velde et al., 2021) have demonstrated the practical relevance and effectiveness of behavioural interventions. These reviews commonly report positive outcomes—particularly from reminder-based and multi-component interventions. However, there is limited analysis of how the concept of nudge is operationalized in the cancer screening literature, how different nudging strategies have been applied to increase cancer screening uptake, and what evidence exists regarding their effectiveness.

This study addresses this gap by conducting a scoping review of empirical studies on nudge interventions aimed at increasing cancer screening participation. In doing so, it clarifies which types of nudges have been tested, how they have been implemented, and what effects have been reported. Specifically, the review aims to explore the current evidence base on the reported effectiveness of nudging to increase the uptake of cancer screening. The findings are intended to provide a foundation for future systematic reviews and may support evidence-informed policymaking and guide the design of future behaviourally informed screening interventions.

## 2. Materials and methods

The present scoping review follows the PRISMA-ScR framework (Tricco et al., 2018). This methodology was chosen as the study intended to map the breadth and characteristics of the available evidence, describe how nudges have been applied across studies, identify relevant conceptual factors, and highlight existing knowledge gaps. These aims align with the strengths of the scoping review approach (Aromataris et al., 2024). The diversity of research designs, combined with the anticipated heterogeneity of the available evidence, precluded the possibility of conducting a formal meta-analysis and substantially limited the extent to which a full data synthesis could be undertaken.

However, to enhance the credibility and analytical depth of the findings, supplementary information on the risk of bias (RoB) in the included studies was assessed—an element typically performed in systematic reviews (Page et al., 2021), though also a possible component within scoping reviews (Aromataris et al., 2024). These additional steps were undertaken to map the methodological quality of the included empirical studies and to strengthen transparency, rigour, and interpretability, while maintaining the scoping review as the primary methodological framework.

For this scoping review, a protocol was developed internally before data extraction, but formal pre-registration was not performed.

### 2.1. Search strategy and selection criteria

Literature search was conducted with the scope to identify studies that represent behavioural economics nudge interventions tested to determine interventions that could increase the uptake of cancer screening, including underlying factors like intention to screen or acquisition of information regarding cancer screening. Our scoping review aimed to map studies that explicitly self-identified their intervention as a ‘nudge’, in order to examine how the concept of nudge is operationalized within the cancer screening literature and to ensure conceptual clarity by focusing on studies using this label.

Based on this, a computer search including ‘nudge’, ‘nudging’ OR ‘nudg*’ AND ‘cancer screening’ was conducted in Scopus, Web of Science, Science Direct, and EBSCO (including Academic Search Complete, Health Source: Nursing/Academic Edition, and MEDLINE) databases/search platforms. The search strategy targeted research articles containing selected terms in the title, abstract, and/or keywords. For detailed search queries in each database, see Appendix B.

**Figure 1.**
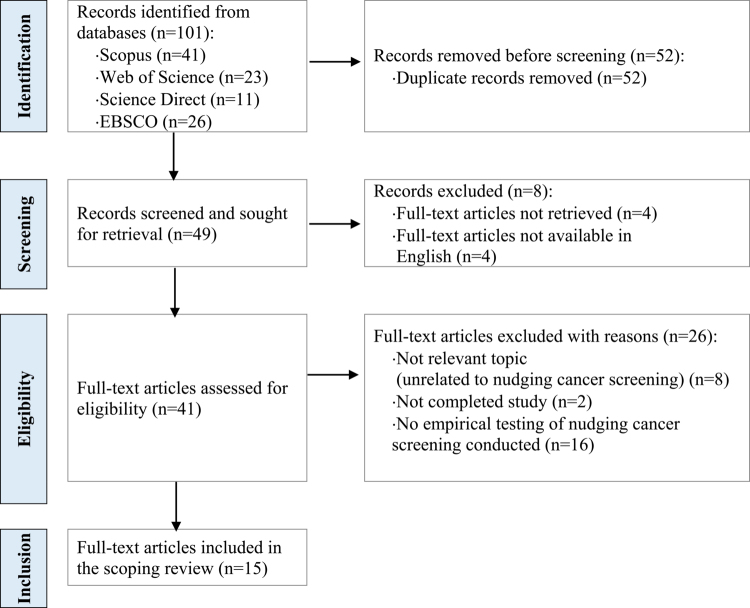
PRISMA flow diagram illustrating identification of studies via databases/search platforms and study inclusions through the stages of the review.

Studies published between January 2008 and August 2025 were considered eligible for inclusion. Records before January 2008 were not included, as the specific conceptualization of the term ‘nudge’ was only established following the publication of Thaler and Sunstein’s book *Nudge: Improving Decisions About Health, Wealth, and Happiness* in 2008. Records after August 2025 were not included as the study selection from databases was conducted on 19 August 2025, following an earlier preliminary analysis performed on data extracted on 29 March 2024. This updated search ensured that the review reflects the most current state of the literature. Exclusion criteria consisted of (1) articles without full-text available, (2) articles without full-text available in English, (3) studies unrelated to nudging cancer screening, (4) not completed studies, and (5) studies that did not conduct empirical testing of nudging interventions designed to promote cancer screening. Following the inclusion/exclusion criteria, empirical studies that quantitatively addressed nudge-related changes in cancer screening uptake were included in the scoping review, irrespective of the methodological quality of the studies. Due to the limited number of sources found via the selection process, no additional quality-based or content-based exclusions were performed. The PRISMA flowchart selection process is illustrated in Figure 1. Selection of studies and data extraction were performed by the first author, and the accuracy of the information was validated by the second author. There were no disagreements between the authors during study selection.

### 2.2. Data charting and synthesis

#### 2.2.1. Data charting

Studies that met our inclusion criteria were assessed by the first author, who extracted various descriptive characteristics (study type, measured effect, country, application context, nudge intervention performer, setting, communication channel, population), information about the nudge intervention tested, and results regarding the effects of the interventions. The second author reviewed the data charting process for accuracy. Any disagreements between the reviewers during data charting were resolved through discussion until consensus was reached. A summary of the attributes of included studies is presented in Appendix C.

#### 2.2.2. Coding of nudge interventions

To verify whether the interventions included in the studies could be considered nudges, we applied the definition proposed by Thaler and Sunstein (2008). Interventions explicitly described as nudges by the original authors were assessed for their conformity with this definition. Additionally, if an intervention was not labelled as a nudge but met the nudge definition and corresponded to a specific nudge type, it was also considered a nudge. Thus, authorial use of the term ‘nudge’ served as a starting point but was not treated as a sufficient criterion for classification.

Some interventions required interpretative judgement when determining whether they fell within the conceptual boundaries of a nudge. Interventions that included small financial incentives or subsidies were classified as nudges when the economic component was modest and primarily served to alter choice architecture rather than to provide substantial compensation (e.g. the micro-incentive intervention by Gupta et al. (2016) and the subsidy in Fukuyoshi et al. (2021)). Similarly, in Schwartz et al. (2017), all intervention arms were considered nudges, including those not explicitly labelled as such by the authors.

To classify nudges tested to increase the cancer screening uptake, an intervention taxonomy based on Münscher et al. (2016) was used (see Appendix A). Based on the descriptions of intervention design presented in the articles, intervention types were determined, and respective codes were allocated. The coding of nudge interventions was conducted by the first author and subsequently discussed with the second author until consensus was reached. As intervention types were not always specifically defined in the reviewed studies, the attribution of a nudge intervention codes was based on resemblance to the psychological mechanisms involved in the intervention. For example, in the case of reminders, even if the intervention was not a typical simple reminder, if it influenced participants by reminding them about cancer screening, the reminder code (A1) was assigned. Similarly, information translation intervention code (I1) was assigned to cases where interventions included simplified messages about cancer screening, while making information visible intervention code (I2) was applied when additional information about cancer screening was provided (both were common but often not specifically defined features in intervention design). Variation in attributing intervention codes is possible, particularly in cases where the methodology is not detailed and/or the intervention sample is not explicitly described.

#### 2.2.3. Effect measures and synthesis methods

The nudge intervention effect was extracted from studies by assessing screening uptake as the primary outcome. If unavailable, alternative measures—in two cases, intention to screen, and in one case, click-through rates for cancer-related information—were used. Firstly, raw results from the studies were extracted (see Appendix C). Then, to enhance comparability, the intervention effects were further standardized as percentage point (pp) increases, compared to a control group where available or to reference values reported in the studies. (The transformations are presented in Appendix D.) This approach aimed to improve the comparability and interpretability of findings across diverse study designs and reporting formats. Finally, effects were summarized in a tabulated overview. Due to the small number of studies and their heterogeneity, the results were synthesized descriptively. Primary focus was given to interventions that showed some effect.

### 2.3. Risk of bias assessment

Both authors assessed each study for potential RoB using the Mixed Methods Appraisal Tool (MMAT) developed by Hong et al. (2018). Based on the MMAT classification, the included studies comprised 10 quantitative randomised controlled trials (RCTs), 3 quantitative non-randomised studies (NRS), and 2 quantitative descriptive studies (QDS). Each study was evaluated against seven methodological criteria specific to its study design. Studies that achieved a score of at least 70% (i.e. meeting 5 out of 7 relevant criteria) were classified as having acceptable methodological quality. Those scoring below 70% (fewer than 5 of 7 criteria) were considered to carry a high RoB. Although the MMAT does not provide a scoring algorithm or recommend cutoffs for categorising studies by RoB, the 70% threshold was applied as a pragmatic criterion to distinguish studies with substantial methodological limitations from those meeting a higher proportion of methodological quality criteria. The use of this threshold aligns with previous research on behavioural economic interventions, which has applied a similar cutoff when using the MMAT to distinguish between studies of relatively higher and lower methodological quality (Werner et al., 2023). An overview of the appraisal results is presented in Appendix E, with a detailed analysis provided in Appendix F.

Following the MMAT guidelines, studies with a high risk of bias were not excluded from this scoping review to ensure a comprehensive mapping of the existing literature, facilitate identification of evidence on effectiveness, and highlight gaps in current research. Furthermore, given the limited number of studies meeting the inclusion criteria, it was deemed important to retain studies that, despite a high risk of bias, may still offer valuable insights.

However, study design and MMAT quality ratings were explicitly reported in the results section, and for analytical purposes, all studies in the tabulated overview were categorized into two groups:

1. RCTs with acceptable RoB—studies that provide the strongest evidence for evaluating the effectiveness of nudging interventions.
2. Other studies—RCTs with high RoB and NRS or QDS, whose results should be interpreted with caution.

### 2.4. Ethics statement

This study did not involve human participants or data, and therefore, ethical approval or exemption from an Institutional Review Board/Ethics Committee was not required.

## 3. Results

### 3.1. Selection of studies

Figure 1 illustrates the PRISMA flowchart selection process. The search strategy produced 101 relevant records overall, i.e. 41 on Scopus, 23 on Web of Science, 11 on Science Direct, and 26 on EBSCO (including Academic Search Complete, Health Source: Nursing/Academic Edition, and MEDLINE). After removing duplicate records, 49 research articles were screened, and 8 research articles were excluded as full-text articles were not retrievable without charge from any of the databases, publishers, or other online sources, or full-text articles were not retrievable in English.

Subsequently, a full-text assessment of these 41 filtered articles revealed that 8 articles were unrelated to the topic of nudging cancer screening, 2 articles presented not completed studies (study protocol/design), and 16 articles did not include empirical research testing nudging cancer screening. After excluding these studies, overall 15 articles providing empirical research examining nudging cancer screening were included in the final analysis.

Results are first presented by describing the study characteristics and the types of nudging interventions applied, followed by the core objective of this review—an analysis of the evidence on the effectiveness of nudge interventions.

### 3.2. Basic study characteristics

An overview of basic study characteristics is in Table 1. Characterizing the studies based on the strength of evidence delivered by their design, most studies (*n* = 9) are RCTs with an acceptable RoB (marked in green in the RoB overview in Appendix E). These studies were conducted using rigorous experimental designs—controlled experiments with comparable control groups and statistical significance testing. The remaining studies (*n* = 6) include RCTs with a high RoB, as well as NRS or QDS. These encompass uncontrolled intervention studies, observational studies, studies comparing results to historical references, and/or studies lacking statistical testing.

**Table 1.** An overview of basic study characteristics.

Dimension	Characteristic
Studies based on strength of evidence [15]	RCTs with acceptable RoB [9], and other studies [6] (including RCT with high RoB[1] and NRS [3] or QDS [2])
Measured effect [15]	Cancer screening uptake [12], intention to screen [2], and click-through rate for provided information regarding cancer [1]
Application context [22]	Cancer screening by localisation: colorectal [9], cervical [5], breast [4], prostate [1], liver [1], lung [1], stomach [1]
Countries [15]	UK [4], US [3], Japan [2], Israel [1], Italy [1], Latvia [1], Malta [1], Norway [1], Saudi Arabia [1]
Settings and communication channel [20]	Conventional, digital and mixed settings: - conventional setting using mail [5], call [3], direct communication [2] - digital setting using SMS [4], e-mail [3], mobile app [1], online survey experiments [2], and digital information materials [1].
(Perceived) nudge intervention performer [15]	Health authorities: health care professionals, health system providers, study team members in healthcare facilities [13], and unspecified (in online survey experiments) [2]

Note: Frequency in square brackets.

The measured effect of interventions typically is cancer screening uptake (*n* = 12), less often underlying factors like intention to screen or acquisition of information regarding cancer screening. Most of the studies included in the review test nudging to screen for colorectal (*n* = 9), cervical (*n* = 5), and/or breast (*n* = 4) cancer, and almost half of the studies (*n* = 7) are performed in the UK and the US. Conventional, digital, and mixed settings are used, with communication channels including mail, SMS, calls, and email. Nudges are mainly delivered (or perceived to be delivered) by health authorities (*n* = 13): health system providers through screening programs, healthcare professionals primarily in primary care, or study team members in healthcare facilities. (Full study characteristics are reported in Appendix C.)

### 3.3. Nudge interventions

Table 2 presents an overview of the empirically tested nudge intervention types identified in this study, including illustrative examples. Across the included studies, interventions typically combined several nudging components rather than relying on a single technique. The most tested intervention was decision assistance by providing a reminder combined with some information provision nudges. Information provision was present in all selected studies, and we considered reminders to be an underlying aspect of the information provision activities used in these interventions.

**Table 2.** Empirically tested nudge techniques to increase cancer screening uptake.

Nudge techniques	Examples
**Decision assistance**	
(A1) Providing reminders [15]	- Mailed invitation, call, direct communication or e.g. SMS ‘*Your cervical smear test is due. To book please call [phone number]’.*
**Information provision**	
(I1) Translation [13]	- Providing loss-frame e.g. ‘*Mortality rate is 40% if not detected early’.*
	- Providing gain-frame e.g. ‘*Cervical cancer screening saves 4500 lives in England every year’.*
	- Simplifying message.
(I2) Making visible [13]	- Providing feedback regarding non-attendance e.g. ‘*Unfortunately, you have not responded to previous invitations’.*
	- Providing quantitative information.
(I3) Providing social reference point [9]	- Providing social norm by *e.g.* *‘6 out of 10 people invited to participate in the screening program accept the invitation’.*
	- Communicating information via primary care practitioner as credible messenger e.g. including their name in SMS.
**Changing decision structure**	
(S2) Changing option-related effort [3]	- Appointment time offered with no need for invitation letter, provision of clear instructions, mailed fecal immunochemical test.
(S1) Changing choice defaults or introducing active choice [2]	- Enhancing individuals to choose between options e.g*.* ‘*Yes, I want to participate and get screened for colorectal cancer to reduce the risk of suffering from this cancer later and to benefit from the effectiveness of early treatments in case of having this c*ancer’ and ‘*No, I would not like to participate in the colorectal cancer screening programme, even if it makes later medical treatments in case I should have this cancer more difficult’.*
(S4) Change option consequences [2]	- Financial microincentives or slight reduction of costs.
(S3) Change range or composition of options [1]	- Examination appointment offerings for women including male practitioners as decoy.

Note: Nudge technique codes in round brackets, frequency in in square brackets. None of the studies included nudge technique *(A2) facilitate commitment.* For technique *(S1) changing choice defaults or introducing active choice* no default option examples retrieved, and only soft version of active choice included in studies (stronger version would be forced choice enhancing individuals to choose between options before progressing to the next stage of the process).

Information provision nudges were implemented in various ways, including information translation by simplified messages (e.g. Fukuyoshi et al., 2021) and provision of loss-frame or gain-frame of cancer screening (e.g. Huf et al., 2020; Maltz et al., 2024; Mizota & Yamamoto, 2021) as well as making information visible by giving feedback regarding non-attendance (e.g. Gorini et al., 2023) or adding additional quantitative information regarding risks and benefits (e.g. Schwartz et al., 2017). These findings indicate that information-based components commonly form a central part of nudging strategies aimed at increasing screening uptake.

Results also indicated a consistent use of social reference points across several studies, either by signalling that participation is considered a social norm with the majority participating (Gorini et al., 2023; Mizota & Yamamoto, 2021; Schwartz et al., 2017; Stoffel et al., 2019) or utilising that invitation is from health authorities (e.g. health professionals) as credible messengers (Huf et al., 2020; Maltz et al., 2024; Mizota & Yamamoto, 2021; Potter et al., 2021; Savicka & Circene, 2020). Few studies employed modifications to decision structure by decreasing the effort needed to participate in cancer screening (Gupta et al., 2016; Mizota & Yamamoto, 2021; Savicka & Circene, 2020), slightly changing consequences of attendance (Fukuyoshi et al., 2021; Gupta et al., 2016), introducing active choice (Stoffel et al., 2022), and changing range or composition of options (Stoffel et al., 2020).

In several studies, nudge interventions involved resource-demanding activities like direct and tailored communication by the health provider and flexibility of the time of screening appointments (Savicka & Circene, 2020), decision-aid presentation in primary care settings (Schwartz et al., 2017), a mobile app with gamified educational content (Orumaa et al., 2022), financial microincentives (Gupta et al., 2016), or full financial subsidy for screening that was not prior fully-subsidised (Fukuyoshi et al., 2021).

### 3.4. Intervention effect

A tabulated overview of the results, including both raw results and intervention effects standardized as pp increases, is provided in Table 3. (See Appendix C for raw results and Appendix D for transformations into standardized expressions of pp changes).

**Table 3.** The overview of studies, applied nudge interventions, and results regarding their effect.

Source	Type of study design, RoB score	Country	Application context: cancer screening by localisation	Nudge intervention	Nudge technique codes^1^	Raw results on intervention effects	Intervention effects standardized as pp increases^2^; general conclusion
**1. RCTs with acceptable RoB**							
**1.1. Intended effect** ^3^							
Gorini et al. (2023)	RCT; RoB 6/7 (acceptable)	Italy	Colorectal	Invitation letters to screening providing normative feedback that invited person did not participate and/or social context that minority did not participate	A1, I1, I2, I3	Screening participation rates were 5.3% in the control condition with standard invitation letter, 7.0% with normative feedback of not participation, 8.2% with minority norm (info that only minority did not participate), and 7.4% with both normative feedback of not participation and minority norm included (*p* = 0.002). Invited subjects in the minority norm arm were more likely to participate (aOR: 1.38; 95% CI: 1.13–1.68).	Increase in cancer screening uptake by 1, 7pp with feedback of not participation, 2, 9pp with minority norm, 2, 1pp with both feedback of not participation and minority norm included compared to standard invitation letter.
Huf et al. (2020)	RCT; RoB 6/7 (acceptable)	UK	Cervical	SMS reminders to book screening that nudge by including social reference point (primary care practitioners name/descriptive norm of attendance) or loss-framed or gain-framed messages	A1, I1, I2, I3	In Study 1 participation was statistically significantly higher in the study arm that included name of primary care practitioners in SMS (31.4%) compared to control with no SMS (26.4%, aOR, 1.29, 95% CI: 1.09–1·51; *p* = 0.002. In Study 2 participation was highest in the study arm that included name of primary care practitioners in SMS (38.4%) and in the study arm that used standard SMS (38.1%) compared to control with no SMS (34.4%), (aOR: 1.19, 95% CI: 1.03–1.38; *p* = 0.02 and aOR: 1.18, 95% CI: 1.02–1.37; *p* = 0.03, respectively).	Increase in cancer screening uptake by 4-5pp compared to no SMS. SMS reminders including name of primary care practitioners specifically yielded the largest improvements in uptake.
Fukuyoshi et al. (2021)	RCT; RoB 6/7 (acceptable)	Japan	Liver^4^	Reminders to promote the hepatitis virus screening rates at worksites targeting employees with simplified messages and information about discounted or fully subsidized cost	A1, I1, S4	The screening rate was 21.2% with standard reminder, 37.1% with nudge-based reminder, and 86.3% with nudge-based reminder including also full subsidy (without copayment of JPY 612). The risk ratio for group with nudge-based reminder was 1.75 (95% CI: 1.45–2.12) and that of group with nudge-based reminder including also full subsidy was 4.08 (95% CI: 3.44–4.83). Incremental cost-effectiveness ratio (ICER), expressed in terms of JPY per one additional person screened was JPY 1168.7 for full subsidy compared to JPY 172.5.for nudge-based reminder.	Increases in screening uptake 65.1 pp for the reminder highlighting the discounted price and including a full subsidy, and 15.9 pp for the reminder highlighting the discounted price without full subsidy, both compared to the simple reminder. The cost-effectiveness higher for reminder without full subsidy.
Stoffel et al. (2019)	RCT (online survey experiment); RoB 6/7 (acceptable)	UK	Colorectal^5^	Verbal quantifiers in communication indicating social reference point of cancer screening uptake	A1, I1, I2, I3	Verbal quantifiers increased screening intentions compared with the control group: from 7.8 to 12.5%, aOR 1.72; 95% CI 1.00–2.96 in the case of ‘a large number’ and to 14.3%, aOR 2.02; 95% CI 1.20–3.38 for ‘nearly half’. Simply communicating that 43% do the test, however, had no impact on intentions (9.9% vs. 7.8% aOR 1.25; 95% CI 0.73–2.16).	Increase in cancer screening intentions by 4.7pp when formulation ‘a large number’ was used and by 6.5pp when formulation ‘nearly half’ was used compared to control group that did not contain any information on uptake.
Stoffel et al. (2020)	RCT (online survey experiment); RoB 6/7 (acceptable)	UK	Colorectal^5^	Examination appointment offerings for women including male practitioners (endoscopists) as decoy	A1, I1, S3	Women were more likely to choose the appointment with the female endoscopist if they were also offered the decoy (the appointment with the male practitioner) (49.3% vs. 25.3%, OR 2.87, 95% CI: 1.76–4.67, *p* < 0.001).	Increased intention/choice to be screened with female practitioner in the presence of the decoy of men practitioner by 24pp compared to an appointment with a female practitioner.
**1.2. Mixed results**							
Gupta et al. (2016)	RCT; RoB 6/7 (acceptable)	US	Colorectal	Mailed fecal immunochemical test (FIT) outreach, automated telephone reminders and financial microincentives ($5 or $10 gift card for returning the test kit)	A1, S2, S4, I2	FIT completion was 36.9% with vs. 36.2% without any financial incentive (*p* = 0.60) and was also not statistically different for the $10 incentive (34.6%, *p* = 0.32 vs. no incentive) or $5 incentive (39.2%, *p* = 0.07 vs. no incentive) groups.	Cancer screening uptake not statistically significantly influenced by financial microincentives compared to simply provision of mailed test. FIT completion 36.2% with FIT outreach without any financial incentive. *(Due to the lack of control group without FIT outreach, standardization to pp increases not possible.)*
Schwartz et al. (2017)	RCT; RoB 6/7 (acceptable)	US	Colorectal	Decision-aids in primary care sites including quantitative information and/or nudge towards stool testing providing encouraging messages with social context and addressing those who wish to procrastinate	A1, I1, I2, I3	Patients who viewed the quantitative module had statistically significantly higher screening uptake (39%) than those who received only basic information (27%, *p* = 0.012). In contrast, patients who viewed the nudge-based information, which included a social norm supporting stool testing, did not exhibit a statistically significant difference in screening uptake compared with the control group who received only basic information.	Increase in cancer screening uptake for patients that viewed the quantitative information by 12pp compared to those who had basic info, while nudge-based social norm information does not provide statistically significant effect.
Stoffel et al. (2022)	Malta	Colorectal	Letters with information about colorectal cancer screening questioning interest in receiving a free fecal immunochemical test (FIT) test providing limited (yes/no) response options and framing of those responses	A1, I1, I2, S1	Enhanced active choice increased acceptance among men by 4.6 pp (50.8% vs 46.2%; OR 1.20; 95% CI: 1.05–1.37, *p* = 0.006), which translated to a statistically significant 3.4 pp increase in participation (42.9% vs 39.5%, aOR 1.15; 95% CI: 1.01–1.31, *p* = 0.040). Among women, enhanced active choice did not affect acceptance or participation; 48.4% of women accepted the invitation in the control condition, while 47.3% accepted it in the enhanced active choice condition (*p* = 0.502).	Increase in cancer screening uptake by enhanced active choice among men by 3.4pp compared to standard invitation with opt-in strategy, but no statistically significant effect among women.	
**1.3. No statistically significant effect**							
Maltz et al. (2024)	RCT; RoB 6/7 (acceptable)	Israel	Colorectal, breast, cervical^6^	Digital messages (emails/SMSs) using message framings on the uptake rates of medical check-ups (gain-framed, loss-framed, physician-recommended, linking to future, or emphasizing personal responsibility)	A1, I1, I2, I3	No statistically significant effect of message framing on uptake rates of medical check-ups compared to neutrally framed messages.	No statistically significant effect of message framing on uptake rates of medical check-ups compared to neutrally framed messages.
**2. Other studies—RCTs with high RoB and NRS or QDS**							
**2.1. Intended effect** ^3^							
Bucher et al. (2022)	QDS (intervention study); RoB 6/7 (acceptable)	US	Breast	Reinforcement learning–enabled emails incorporating nudges suggesting to attend mammography	A1, I1, I2, I3	Cancer screening uptake of 24.99% scheduled and 22.02% attended to screening of patients who were overdue.	Cancer screening uptake of 24.99% scheduled and 22.02% attended to screening of patients who were overdue. *(Due to the lack of control group, standardization to pp increases not possible.)*
Elfakki et al. (2024)	RCT; RoB 3/7 (high)	Saudi Arabia	Colorectal	Direct positively framed health professional invitation to screening (family physicians and nurses in the intervention arm received on‑site nudging training: received recommendations regards the use of SMS and calls, and invitation language)	A1, I1, I2	Cancer screening uptake higher in the two intervention sites (38% and 26%), than in the two control sites (18% and 18%).	Increase in cancer screening uptake by 8-20pp compared to routine care.
Mizota and Yamamoto (2021)	NRS (intervention study) RoB 5/7 (acceptable)	Japan	Colorectal, breast, lung, cervical, stomach	Leaflets with colorectal, breast, lung, cervical, and stomach cancer screening recommendation materials including messages containing combination of nudges (additional and framed information, clear instructions, social reference)	A1, I1, I2, S2, I3	Overall screening uptake improved by 2.6pp or 1.44 fold (*p* < 0.001) compared to historical control: by 1.7pp or 1.38 fold (*p* < 0.001) for colorectal, by 3, 0pp or 1.45 fold (*p* < 0.001) for breast, by 2.1pp or 1.32 fold (*p* < 0.001) for lung, by 3.5pp or 1.50 fold (*p* < 0.001) for cervical, by 2.2pp or 1.53 fold (*p* < 0.001) for stomach cancer screening.	Increase in cancer screening uptake by 2.6pp compared to historical control (by 1.7pp for colorectal, by 3,0pp for breast, by 2.1pp for lung, by 3.5pp for cervical, by 2.2pp for stomach cancer screening).
Orumaa et al. (2022)	NRS (retrospective cohort study); RoB 7/7 (acceptable)	Norway	Cervical	Gamified educational cancer and its prevention related content via mobile app	A1, I1, I2	6 months after enrolment 29.6% of the women in the intervention group and 15.21% of those in the comparable historical control group underwent a cervical exam (*p* < 0.01). Women exposed to the app were 2 times more likely to attend screening (adjusted HR 2.3, 95% CI 2.0-2.7).	Increase in cancer screening uptake by 14.4pp for app users 6 months after enrolment compared to comparable historical control group not exposed to app.
Potter et al. (2021)	NRS (intervention study); RoB 3/7 (high)	UK	Prostate	Targeting black men or their wives/girlfriends via email with a picture of a black doctor suggesting prostate cancer screening for men, providing information about the risks, and including a link to find more information	A1, I2, I3	The click-through rate was 15.5% for women and 38.5% for men, both higher compared to the historical control - the standard Prostate Cancer UK email click-through rate of 6.3%	Increased click-through rate for provided information regarding cancer for man by 32.2pp and for woman by 9.2pp compared to Prostate Cancer UK click-through rate.
Savicka and Circene (2020)	QDS (intervention study); RoB 2/7 (high)	Latvia	Breast, cervical	A direct offer by the healthcare provider call centre to perform breast and cervical cancer screening at a specific time without the need for a letter of invitation (those who did not pick up received SMS)	A1, S1, S2, I1, I2, I3	Breast cancer screening uptake increased from 34% in the pre-intervention year (2016) to 72% in the post-intervention year (2018), and cervical cancer screening uptake increased from 24% in 2016 to 78% in 2018.^7^	Increase in cancer screening uptake by 38pp for breast cancer and by 54pp for cervical cancer screening (post intervention year 2018 compared to pre-intervention year 2016).

Notes:

^1^ Categorisation based on adapted taxonomy of choice architecture categories and techniques by Münscher et al. (2016); see Appendix A.

^2^ Increase in cancer screening uptake, intentions, or click-through rate for provided information at least to some extent.

^3^ To enhance comparability, results transformed to pp increase. The effect is derived from statistically significant results. If statistical significance was not tested, the difference between intervention results and the provided reference was reported. A detailed effect estimate in original studies, their precisions, and transformation to pp are available in Appendix D.

^4^ Hepatitis virus screening itself is not labelled as cancer screening, but it helps to identify people at higher risk of liver cancer, allowing for closer monitoring and potentially early detection of liver cancer.

^5^ Flexible sigmoidoscopy screening is commonly referred to as bowel scope screening.

^6^ Different health check-ups, including colorectal and breast cancer screenings, and human papillomavirus (HPV) important for decreasing cervical cancer.

^7^ These data were not explicitly reported in the original study by Savicka & Circene (2020), as the study did not aggregate the results. However, the figures were calculated based on the information provided in Table 4 of the same publication.

#### 3.4.1. Overall effectiveness

Reviewed studies generally demonstrate the potential of nudging to increase the uptake of cancer screening. Most (*n* = 14) studies have the intended effect of an increase in cancer screening uptake, screening intentions, or click-through rate for provided cancer-related information at least to some extent for some intervention types or tested subgroups (see Table 3, Sections 1.1 and 2.1 of the table for studies demonstrating the intended effect, and Section 1.2 of the table for mixed results). The effect of nudge interventions varies widely - from no statistically significant impact (Maltz et al., 2024), or small effects of a few pp (e.g. 2–5 pp in Gorini et al., 2023; Huf et al., 2020; Mizota & Yamamoto, 2021), to substantial effects of several tens of pp (e.g. up to 54 pp in Savicka & Circene, 2020, and up to 65.1 pp in Fukuyoshi et al., 2021). Results differ case by case based on study design, settings, and combination with other resource-demanding activities.

As interventions are performed by combining different nudge techniques and in some cases involve resource-demanding activities, only the combined effect can be observed, and it is not possible to scale down the impact of each nudge technique. However, studies generally indicate promising results regarding the nudging effect to increase the uptake of cancer screening.

Distinguishing studies based on their methodological approach indicates that results from RCTs with acceptable RoB (*n* = 9; listed under Section 1 in Table 3) demonstrate positive effects of nudging (*n* = 5), mixed results (*n* = 3), and no statistically significant effect (*n* = 1). The results of these studies can be considered highly credible. Other studies—RCTs with high RoB and NRS or QDS (*n* = 6; listed under Section 2 in Table 3)—all show positive results. However, in these studies, the strength of evidence is compromised by methodological issues—a high RoB for RCTs, studies applying uncontrolled interventions, observational designs, comparisons with historical reference groups, and/or lacking statistical significance testing.

#### 3.4.2. Reminders and informational nudges

To examine the effectiveness of reminders and informational nudges in promoting cancer screening, studies employing interventions composed exclusively of these nudge techniques—specifically those coded as A1, I1, I2, and/or I3 (Bucher et al., 2022; Elfakki et al., 2024; Gorini et al., 2023; Huf et al., 2020; Maltz et al., 2024; Orumaa et al., 2022; Potter et al., 2021; Schwartz et al., 2017; Stoffel et al., 2019)—were analysed. These studies have demonstrated the potential to increase cancer screening uptake by at least a few pp.

In the RCT *(acceptable RoB)* conducted by Gorini et al. (2023) in Italy, invitation letters for colorectal cancer screening that included normative feedback—informing recipients that they had not participated and/or that only a minority of others had participated—resulted in a 1.7 to 2.6 pp increase in uptake compared to a standard invitation letter. In line with these findings, an RCT *(acceptable RoB)* conducted as an online survey experiment in the UK by Stoffel et al. (2019) demonstrated that including social reference information in messaging statistically significantly increased intentions to participate in colorectal cancer screening—by 6.5 pp when using the phrase ‘nearly half participate,’ and by 4.7 pp when using ‘a large number participate,’ compared to a control group that received no uptake information. Similarly, in the UK, an RCT *(acceptable RoB)* by Huf et al. (2020) found that SMS reminders containing informational nudges—such as messages emphasizing social norms or using gain/loss framing—led to a 4–5 pp increase in cervical cancer screening uptake compared to no SMS reminder.

However, not all RCTs with acceptable RoB demonstrated consistently positive results. For example, the study by Schwartz et al. (2017) reported mixed findings. The interventions tested among primary care patients in the US included basic information, quantitative information, and/or a nudge toward stool testing with the fecal immunochemical test, framed as a socially normative choice. While the authors labelled only the social-context message as a nudge, we consider all interventions examined in the study to be consistent with the definition of a nudge. The study found that messages emphasizing social context did not statistically significantly increase colorectal cancer screening uptake compared to basic information alone. In contrast, quantitative decision aids provided in primary care led to a 12 pp increase in uptake. Similarly, the RCT *(acceptable RoB)* by Maltz et al. (2024) in Israel found no statistically significant effect of message framing in emails or SMSs on the uptake of medical check-ups—including colorectal, breast, and cervical cancer screenings—when compared to neutrally framed messages.

In contrast to the more moderate, mixed, or statistically non-significant effects observed in RCTs with acceptable RoB, evidence from less methodologically robust studies suggests that reminders and informational nudges may lead to even higher uptake rates across diverse populations and screening settings. For instance, in the UK, an intervention study by Potter et al. (2021) targeted underserved black men using culturally tailored emails from community organisations. These emails provided information about prostate cancer risks and featured a picture of a black doctor recommending screening. The click-through rate for accessing cancer-related information exceeded the standard Prostate Cancer UK benchmark by 32.2 pp among men, and by 9.2 pp among their wives or girlfriends. Similarly, Bucher et al. (2022) in an intervention study in the US used reinforcement learning-enabled emails with nudges to promote mammography uptake among overdue patients. As a result, 24.99% of recipients scheduled a screening and 22.02% attended. However, due to the absence of a control group, the results are not comparable to other studies, as the effect size cannot be expressed in terms of a pp increase. Moreover, the lack of a control group makes it unclear whether the observed outcomes can be directly attributed to the intervention. Another novel approach was demonstrated in Norway, where a retrospective cohort study by Orumaa et al. (2022) showed that a gamified educational mobile app containing content related to cancer and its prevention increased cervical cancer screening uptake by 14.4 pp compared to a historical control group.

While most of these studies that utilise reminders and informational nudges have focused on indirect messaging strategies, some have examined more direct, interpersonal approaches to increasing screening uptake. In Saudi Arabia, an RCT *(high RoB)* by Elfakki et al. (2024) evaluated the impact of training healthcare professionals to deliver direct, positively framed invitations to colorectal cancer screening. Screening uptake was 8-20 pp higher in intervention sites compared to control sites, where professionals had not received training and continued to provide routine care.

Overall, the effect of reminders and informational nudges appears to vary. RCTs with acceptable RoB show modest effectiveness—up to a 5 pp increase in cancer screening uptake (Huf et al., 2020) and up to a 6.5 pp increase in screening intentions (Stoffel et al., 2019)—while others report mixed or statistically non-significant effects (Maltz et al., 2024; Schwartz et al., 2017). In contrast, studies with weaker methodological rigor report substantially larger effects, including up to a 20 pp increase in cancer screening uptake (Elfakki et al., 2024) and up to a 32.2 pp increase in click-through rates to cancer-related information (Potter et al., 2021).

#### 3.4.3. Modifying decision structure

Beyond reminders and informational nudges, several interventions have explored strategies that modify decision structures—such as altering available options, adjusting perceived consequences, or reducing the effort required for action. To examine the effects of such approaches, studies incorporating both informational components (nudge techniques A1, I1, I2, and/or I3) and decision-structuring elements (S1, S2, S3, and/or S4) were analysed (Fukuyoshi et al., 2021; Gupta et al., 2016; Mizota & Yamamoto, 2021; Savicka & Circene, 2020; Stoffel et al., 2020; Stoffel et al., 2022). Results suggest that, although outcomes vary, interventions combining reminders and informational elements with modifications to decision structures have the potential to demonstrate greater effectiveness than purely informational approaches—in some cases achieving increases of several tens of pp.

Highly effective interventions involving changes to decision structure have been applied in an RCT *(acceptable RoB)* by Fukuyoshi et al. (2021). The study was conducted in Japanese worksites to evaluate strategies for increasing uptake of hepatitis virus screening—a key method for identifying individuals at elevated risk of liver cancer—by providing a full subsidy for employee screening costs.

It compared three interventions: (1) a standard reminder requiring a JPY 612 copayment; (2) a nudge-based reminder, also requiring a JPY 612 copayment, but using simplified information and highlighting the discounted price by striking out the original JPY 2040 cost; and (3) a nudge-based reminder with similarly simplified information but emphasising that the screening was fully subsidised by striking out the original JPY 2040 cost. As part of our analytical framework, we consider all three interventions as forms of nudging. The standard reminder, although not explicitly labelled as a nudge, functions as one; likewise, the fully subsidised option can also be considered to remain within nudge boundaries, as waiving the small JPY 612 copayment does not constitute a substantial change in economic incentives. Compared to the standard reminder, the nudge-based reminder combined with a full subsidy yielded the highest effectiveness, increasing screening uptake by 65.1 pp, while the nudge-based reminder without the full subsidy yielded an increase of 15.9 pp. However, the incremental cost-effectiveness ratio was nearly seven times better for the nudge-based reminder alone (JPY 172.5 per additional person screened) compared to the full subsidy (JPY 1168.7 per additional person screened), indicating that offering a full subsidy was the most effective but not the most cost-effective approach.

In another RCT *(acceptable RoB)*—an online survey experiment by Stoffel et al. (2020) – a substantial effect of modifying decision structure without involving financial consequences was demonstrated. In this UK-based study, presenting a male practitioner as a decoy increased women's preference for scheduling a colorectal cancer screening appointment with a female endoscopist by 24 pp. This indicates that subtle changes in how choices are presented, without adding material incentives, can meaningfully influence screening choices.

Other RCTs with acceptable RoB involving decision structure changes demonstrate mixed results. In a study of Stoffel et al. (2022), an enhanced active choice approach—letters prompting a yes/no response to receive a free FIT test in Malta—demonstrated a 3.4 pp increase in colorectal cancer screening uptake among men compared to a standard opt-in invitation, but no statistically significant effect among women. A US-based study by Gupta et al. (2016) demonstrated that adding small financial incentives ($5 or $10 gift cards) to mailed FIT outreach and reminders did not have a statistically significant effect on colorectal cancer screening uptake rates compared to no incentive. FIT completion rates were statistically similar across all groups: 36.2% without any incentive, 39.2% with the $5 incentive, and 34.6% with the $10 incentive. Given the very small monetary amounts involved, we regarded the interventions as remaining within the conceptual boundaries of nudging, since they did not introduce substantial economic incentives. Whether the incentives were too small to yield an effect or there is resistance to incentivizing in a sensitive field like cancer screening requires further examination. While the microincentives did not yield a statistically significant effect, the study revealed a generally high responsiveness to mailed FIT outreach—likely driven by the reduced effort required to complete the test.

Evidence from less methodologically robust studies that combine an informational approach with decision structure changes provides diverse results. In a non-randomized intervention study by Mizota and Yamamoto (2021) in Japan, an intervention primarily based on informational nudging—using leaflets that included multiple elements such as additional and framed information and social references—along with an effort-reducing nudge in the form of clear, step-by-step instructions for undergoing cancer screening, resulted in a 2.6 pp overall increase in cancer screening uptake compared to historical controls. The limited effectiveness may be attributable to the fact that the provision of instructions only slightly reduces the effort required.

In contrast, the study that tested a more substantial reduction in effort alongside the use of an active choice approach reported exceptionally high levels of effectiveness. This was demonstrated in an intervention study by Savicka and Circene (2020) *(high RoB)* in Latvia, where a direct offer from the healthcare provider’s call centre—offering breast and cervical cancer screening appointments without requiring a letter of invitation—was implemented. Screening uptake increased substantially: breast cancer screening rose by 38 pp and cervical cancer screening by 54 pp. Though the study lacked a comparable control group and statistical testing—limiting the quality of evidence—it nevertheless indicated high potential for substantial effort reduction and a specific active choice setting to support screening decisions. This should be further investigated, preferably in well-designed controlled experiments.

Consequently, the reviewed studies suggest that combining reminders and informational nudges with modifications to decision structure has the potential to achieve high levels of effectiveness—for example, up to a 65.1 pp increase in uptake when a full financial subsidy is provided (Fukuyoshi et al. (2021), or up to 54 pp when a direct offer from the healthcare provider is introduced (Savicka & Circene, 2020). However, the quality of evidence across studies varies, and more well-designed RCTs are needed to confirm this.

## 4. Discussion

### 4.1. Findings in the context of prior studies

Our study is the first to synthesise evidence on nudge interventions and their effectiveness in increasing cancer screening uptake, regardless of the communication channel used. A comparison of this review’s findings with prior literature reveals that the identified nudging techniques largely correspond to those outlined in the literature review by Hofmann and Stanak (2018), demonstrating that similar strategies have been repeatedly identified to encourage participation in screening. In line with the findings of a meta-analysis of digital nudge effects on cancer screening by Wang et al. (2025) and other comparable reviews on preventive health behaviors, this study finds generally positive, though heterogeneous, effects of nudging, underscoring both the potential and the variability of such interventions.

Evidence from diverse health prevention domains supports this conclusion. For instance, nudging strategies have been linked to improved vaccine uptake and confidence (Reñosa et al., 2021), including during the COVID-19 pandemic (Zhang & Jin, 2023). Positive effects have been reported in reducing missed healthcare appointments through reminder systems (Werner et al., 2023), as well as in promoting self-management and healthier behaviours among individuals with chronic conditions (Kwan et al., 2020; Möllenkamp et al., 2019). When applied to HIV-related behaviours, nudging has shown some positive effects (Ahmed & McNamee, 2023); however, the evidence remains limited and weak, underscoring the need for more rigorous research—an issue also echoed in this review. Similar to the findings of this review, the study by Lee et al. (2024) on early COVID-19 responses reported overall positive potential of nudge-based interventions, but effectiveness substantially depended on the situational context.

At the same time, a growing body of large-scale research cautions against overestimating the effectiveness of nudging. Interdisciplinary large-scale quantitative reviews and meta-analyses indicate that nudges generally yield modest behavioural effects, with reported effect sizes around Cohen’s d = 0.4 (Beshears & Kosowsky, 2020; Mertens et al., 2022) and median effect sizes of approximately 21% (Hummel & Maedche, 2019). Moreover, a large comparative analysis of RCTs revealed that nudges implemented by academic researchers achieved an effectiveness of 33.4% (8.68 pp), whereas the impact in large-scale government-led Nudge Unit RCTs was considerably smaller—only 8.0% (1.39 pp) (Linos & Della, 2022), indicating a generally modest real-world effectiveness.

In this regard, the present study complements existing evidence and reinforces the notion that, while nudging is a promising approach, its effectiveness should not be overstated and remains highly context-specific. For both research and policy, this underscores the importance of carefully tailoring interventions to the behavioural, cultural, and institutional settings in which they are implemented.

### 4.2. Challenges in analysing the effectiveness of nudging

An initial challenge lies in determining whether the interventions included in the studies qualify as nudges, given the conceptual ambiguity and inconsistent terminology used across the included studies. This challenge can be addressed by assessing each intervention against the conceptual definition of a nudge. Such classification inevitably involves interpretative judgment. Recognising this, applying the term ‘nudge’ as consistently as possible is important for comparing and synthesising the resulting evidence in a meaningful and reliable way.

Another challenge arises from the fact that the sample of studies included in the scoping review, along with differences in study settings, designs, application contexts, and additional influencing factors, does not allow for confident generalisation or unambiguous conclusions about the effectiveness of nudging techniques. Drawing firm conclusions about the effectiveness of specific nudging methods is particularly limited due to the heterogeneity introduced by combining multiple techniques within a single intervention. Studies on nudging to increase cancer screening uptake typically involve interventions in which several nudge techniques are applied simultaneously, making it impossible to isolate the contribution of each component. This reflects a broader trend, as combining different nudging techniques is common practice across studies testing interventions. Similarly, in other behavioural domains, combined interventions are frequently used, with evidence suggesting that nudges tend to be more effective when applied together (e.g. Broers et al., 2017; Webb et al., 2010).

In future research, analysing individual components of interventions—separately and in combination, where such testing is feasible—could help identify which specific nudging techniques are essential and most effective in driving the overall impact. A unified framework for classifying and coding nudges could further support the comparability and synthesis of findings across studies.

### 4.3. Effectiveness across nudging techniques

Although effectiveness varies, nudging has shown potential to increase cancer screening uptake, and the studies included in this review offer indications of how different nudging techniques may vary in their impact. Sole reminders and informational nudges—powered by simplified loss-framed or gain-framed messages, feedback regarding non-attendance, social norm of participation, and health authority as a credible messenger—have demonstrated modest positive effects, typically resulting in a few pp increases in uptake, with a maximum of up to 20 pp (an exceptionally high result reported in an RCT *(high RoB)* by Elfakki et al. (2024)). The effectiveness of providing information and reminders can be attributed to the fact that inaction often results from a combination of factors—such as inertia, procrastination, competing obligations, and forgetfulness—all of which can be addressed through timely reminders (Sunstein, 2014). Importantly, these types of nudges are low-cost, easy to implement (often delivered via SMS, email, or letters), and represent a scalable approach suitable for reaching large populations.

However, in line with nudge intervention ladders, as suggested by Patel et al. (2020), information provision can be considered a light intervention, and to achieve a greater effect, stronger interventions like direct influence on decisions by planning or guiding choices regarding the arrangement and acquisition of cancer screening tests are needed. Indeed, studies utilizing a more paternalistic approach by reducing the effort to get screened and providing direct invitations by health professionals to screening have yielded a higher effect than purely informative approaches. The effectiveness of nudges that, in addition to reminding and informing, also incorporate modifications to decision structures yeald to uptake increase up to several tens of pp, with a maximum of up to 65.1pp—an exceptionally high result reported in an RCT *(acceptable RoB)* by Fukuyoshi et al. (2021). These findings are consistent with the general nudging literature as comprehensive quantitative studies have indicated that nudges tend to be more powerful for driving changes in outcomes when they are targeting decision structure (Mertens et al., 2022) and automating some aspects of the decision-making process (Beshears & Kosowsky, 2020), thus demanding less effort on attention for individuals to make the preferred choice.

Building on this, the intervention ladder proposed by Patel et al. (2020) may serve as a useful framework and policy tool if applied more systematically—for example, by mapping various nudging strategies along its continuum of intrusiveness to evaluate both expected effectiveness and ethical implications.

While these patterns highlight differences in effectiveness across nudging techniques, the type of cancer targeted by a screening programme could plausibly also influence how effective a given nudge is, for example, through differences in baseline uptake, perceived risk, or existing screening pathways. In this scoping review, however, the small number of studies per cancer type and the variability in intervention content and outcome measures did not permit a meaningful comparison of nudge effects across cancer sites. Future studies could explicitly examine whether nudges work differently for different cancer types and in different screening contexts.

### 4.4. Resource-demanding elements in nudging

The effect of nudges seems to be large when nudging involves resource-demanding activities. For instance, an uptake increase by 61.1 pp was observed for an intervention that provides a full subsidy for screening and thus requires considerable financial investment (RCT *(acceptable RoB)* by Fukuyoshi et al., 2021). Likewise, mailing fecal immunochemical test kits directly to individuals has demonstrated ~36 to 39% uptake, but also incurs financial costs (RCT *(acceptable RoB)* by Gupta et al., 2016). Furthermore, direct communication from healthcare providers has demonstrated up to 54pp uptake increase, but it also necessitates dedicated time from health professionals, making it an effort-intensive approach (intervention study by Savicka & Circene, 2020 *(high RoB)*). These examples suggest that while such nudge interventions can substantially enhance screening uptake, they might require considerable investment. Only one study included in the scoping review provided a cost-effectiveness analysis (an RCT *(acceptable RoB)* by Fukuyoshi et al., 2021). The study found that while the combination of a nudge-based reminder and a full subsidy proved to be the most effective strategy, it was not the most cost-effective. In contrast, a standalone nudge-based reminder yielded greater cost-effectiveness, despite lower overall effectiveness. This highlights the need for further cost-effectiveness analyses of nudging interventions aimed at increasing cancer screening uptake to better inform policy decisions.

### 4.5. Involvement of healthcare professionals

Few studies indicated the crucial role of healthcare professionals in communication. In the RCT *(acceptable RoB)* by Huf et al. (2020), which tested different SMS reminders for booking cervical cancer screenings, the reminder that included the name of the primary care practitioner yielded the greatest improvement in uptake, demonstrating a 5 pp increase. In an RCT *(high RoB)* by Elfakki et al. (2024), an 8-20 pp increase in uptake was observed at intervention sites where a direct, positively framed health professional invitation to screening was used, compared to sites with no intervention. The intervention study of Savicka and Circene (2020) *(high RoB)* demonstrated an increase of 38 pp for breast cancer and of 54 pp for cervical cancer screening compared to historical references and linked the direct offer by the healthcare provider to perform screening to high screening uptake rates. Thus, studies indicate that health professionals serve as authorities by which the credible messenger effect could be utilized, particularly when direct communication is used. However, due to the high RoB, lack of comparable control groups and statistical testing in the studies by Elfakki et al. (2024) and Savicka and Circene (2020), confidence in the body of evidence is limited, and the magnitude of the effect from the direct health care provider communication should be further examined in rigorous controlled experiments.

### 4.6. Limitations

This scoping review is limited to studies that explicitly labelled interventions as ‘nudges’ and/or processes as ‘nudging’, which enabled the selection of these studies by those search terms. It is possible that other studies that do not contain the terms ‘nudge’ and/or ‘nudging’ but still encompass nudge-like interventions are not included in this scoping review. Thus, this review should not be considered exhaustive of all behaviourally informed interventions in the cancer screening literature. Because the review focused on studies in which authors themselves identified the interventions as nudges, the search strategy did not incorporate broader behavioural intervention terminology that may conceptually overlap with nudging but is not consistently labelled as such. Including additional search terms would have substantially broadened the scope of the review and shifted its conceptual emphasis from examining how the term nudge is used and operationalized to retrospectively categorizing a wider range of behavioural interventions. Such a broader exploration would be highly valuable, though it would require a separate review with distinct eligibility criteria and methodological considerations.

Another limitation is that the review did not include backward citation searching. This decision was made to preserve conceptual consistency with the inclusion criteria, which required explicit authorial use of the term nudge. Taken together, these limitations reflect deliberate conceptual rather than procedural boundaries.

The study was not preregistered, which we acknowledge as a methodological limitation. However, because the review’s conceptual boundaries and eligibility criteria were explicitly defined and consistently applied, we do not expect this to have influenced the results.

### 4.7. Implications for research

To properly address the policy challenges of sub-optimal cancer screening uptake, further empirical research is required to build robust evidence on the effectiveness of nudging strategies. Current findings reveal a pattern: while studies with acceptable RoB provide diverse results, studies with less rigorous designs tend to report positive effects. To prevent the possibility that inadequately controlled studies may inflate the actual effect of nudging and to achieve a clear and objective understanding of the real impact of these interventions, it is essential to conduct rigorously designed experimental research.

Future research should prioritise well-designed RCTs featuring comparable control groups, statistical significance testing, and transparent reporting. To improve methodological quality and reduce RoB, several enhancements should be implemented: preregistration of study protocols to ensure transparency, randomisation and adequate control conditions to support causal inference, standardised outcome measures, and comprehensive reporting of attrition and response rates. Follow-up assessments are also recommended to evaluate the persistence of effects over time. These practices would mitigate common sources of bias and enhance the comparability and reproducibility of results.

Beyond these foundational methodological improvements, future research should consider study designs that allow for the systematic assessment of individual nudging components. Such approaches include factorial and fractional factorial experiments, as well as Multiphase Optimization Strategy (MOST) frameworks, which enable researchers to estimate the independent and interactive effects of specific intervention elements. Applying these component-level designs would make it possible to identify which nudges contribute most to increasing screening uptake and to develop more efficient, scalable, and evidence-based intervention packages. Research could also be expanded through micro-randomized trials (MRTs) and Sequential Multiple Assignment Randomized Trials (SMARTs), which allow for the evaluation of time-varying and response-dependent intervention effects, thereby supporting the development of adaptive and context-sensitive nudging strategies.

Furthermore, applying rigorous reporting standards is essential. Given the variability in how nudging interventions were described across the included studies, adherence to a minimum set of reporting items would improve the interpretability and comparability of future research. Existing guidelines for intervention reporting—such as TIDieR (Hoffmann et al., 2014) and CONSORT extensions (CONSORT Group, n.d.), including reporting standards specifically for RCTs of social and psychological interventions (Grant et al., 2018)—together with other specialised frameworks developed for particular study designs or relevant to behavioural and choice-architecture interventions, have outlined detailed reporting requirements. These include providing clear descriptions of intervention components, delivery and timing, study design, outcome measures, and evidence of intervention fidelity, among other essential elements. Consistently applying reporting standards would enable researchers to more accurately assess the mechanisms, implementation processes, and effectiveness of nudging interventions. Moreover, offering detailed descriptions and concrete examples of intervention materials would help address features that are, at times, underreported—such as text length, emphasis, and other presentational attributes that may influence effect sizes—thereby strengthening the replicability of studies.

In addition, previous research has shown that results regarding nudge intervention effect should be assessed with caution, considering the selective publication bias in social sciences in general (Franco et al., 2014) and regards nudging in particular (Beshears & Kosowsky, 2020, Hummel & Maedche, 2019, Mertens et al., 2022, Linos & Della, 2022) recognizing the possibility that studies not providing anticipated nudging effect might be published less and thus be unrepresented in reviews.

As this review focused on interventions explicitly labelled as nudges, expanding future reviews with complementary search terms may help identify other promising behavioural strategies. More specifically, incorporating search terms that reflect specific intervention types potentially classified as nudges (e.g. reminders, defaults, feedback) could provide a more comprehensive understanding of the literature. Building on this, the scoping review may serve as foundational groundwork for future systematic reviews—whether those aiming to provide a broader synthesis or those adopting a more targeted focus on particular nudging strategies, specific cancer types, methodological approaches used in the studies, or other context-dependent factors identified through this review.

Moreover, the practical value of nudging interventions would be strengthened by the inclusion of cost-effectiveness analyses. Given resource constraints in public health systems, understanding not just which intervention works, but which has the best cost-effectiveness, is critical for policy uptake and implementation.

## 5. Conclusions

Based on 15 studies, this scoping review demonstrates empirically tested nudging techniques to increase cancer screening uptake. Interventions include reminders as underlying nudge interventions combined with simplified and framed messages, feedback regarding non-attendance, additional information provision, referring to social norms regards participation, using health authority as a credible messenger, providing direct health professional invitations to the appointment, offering microincentives, mailing screening kits, and other interventions.

The review generally indicates a positive effect of nudge interventions to increase the cancer screening uptake and its underlying factors, like intention to screen or acquisition of information regarding cancer screening. Reported effects range from statistically non-significant findings to substantial increases, with the highest recorded effect reaching 65.1 pp. RCTs with acceptable RoB report diverse results, whereas studies lacking rigorous control tend to present positive outcomes. The most effective interventions involved decision structure modifications, such as altering options, adjusting consequences, or reducing effort. Effectiveness also appeared to be higher when interventions included resource-demanding components and/or involved healthcare professionals.

Further studies applying diverse nudges in various settings are needed to expand the evidence base regarding the effectiveness of specific interventions in increasing cancer screening uptake. In particular, building a reliable evidence base requires more RCTs with comparable control groups and robust statistical evaluations.

## Supplementary Material

Supplemental Material Appendices.docx

## Data Availability

The authors confirm that the data supporting the findings of this study are available within the article and its supplementary materials. (Data on the excluded studies are not included in the article, as they are not relevant to the primary findings of this review; however, these details can be provided by the corresponding author, upon reasonable request).
