# Supplementary material for: Nudging to increase the uptake of cancer screening: scoping review of empirical studies
Source: Health Psychol Behav Med. 2026 Mar 3;14(1):2639784. doi: 10.1080/21642850.2026.2639784 (PMC12958388; doi:10.1080/21642850.2026.2639784)
Supplement: Supplemental Material — Appendices.docx [file RHPB_A_2639784_SM8451.docx]

**Appendices**

**Appendix A. Choice architecture categories and techniques**

Based on Münscher et al. (2016), with additions from Mertens et al. (2022) (direct citations included) and with authors’ additions *(in italic).*

| Psychological barrier | Intervention category:  description | Intervention code, intervention technique,  explanation |
| --- | --- | --- |
| Limited access to decision-relevant information | Decision **information**:  increase the availability, comprehensibility, and/or personal relevance of information | ·I1 : Translate information  Adapt attributes to facilitate processing of already available information and/or shift decision maker’s perspective. Includes: reframe, simplify.  ·I2 : Make information visible  Provide access to relevant information. Includes: make own behaviour visible (feedback), make external information visible.  ·I3 : Provide social reference point  Provide social normative information to reduce situational ambiguity and behavioural uncertainty. Includes: refer to descriptive norm, refer to opinion leader, *communicate information via credible messenger* |
| Limited capacity to evaluate and compare choice options | Decision **structure**:  alter the utility of choice options through their arrangement in the decision environment or the format of decision making | ·S1 : Change choice defaults or *introduce active choice*^[[1]](#footnote-1)^  Address behavioural inertia, loss aversion, and/or perceived endorsement by defaults or prompted choice. Includes: set no-action default, use prompted active choice.  · S2 : Change option-related effort  Adjust effort to remove friction from desirable choice option. Includes: increase/decrease physical/ financial effort.  · S3 : Change range or composition of options  Adjust the decision format to facilitate evaluation. Includes: change categories, change grouping of options.  · S4 : Change option consequences  Adapt social consequences or microincentives to address present bias, bias in probability weighting, and/or loss aversion. Includes: connect decision to benefit/cost, change social consequences of the decision, *introduce financial microincentives or slight reduction of costs* |
| Limited attention and self-control | Decision **assistance**:  facilitate self-regulation | · A1 : Provide reminders  Increase the attentional salience of desirable behaviour to overcome inattention due to information overload.  · A2 : Facilitate commitment  Encourage commitment to counteract failures of self-control. Includes: support self-commitment/ public commitment |
| *Note.* Intervention codes introduced with intention to facilitate concrete intervention classification and increase [comparability](javascript:goDic('?q=comparability&g=2%27)). | | |

**Appendix B. Search queries in databases/search platforms for an overview of empirical studies regarding nudging to increase the uptake of cancer screening**

Search conducted on 19.08.2025.

| Database/  search platform^1^ | Search criterion | Articles found |
| --- | --- | --- |
| Scopus | Search words: nudg* AND cancer screening. Search within title, abstract, keywords. Limited to journals. Limited to articles. Limited to year 2008 to 2025. | 41 |
| Web of Science | Search words: nudg* AND cancer screening. Search within abstract. Limited to articles. Limited to year 2008 to 2025. | 23 |
| Science Direct | Search words: nudge AND cancer screening, nudging AND cancer screening. Search within title, abstract, keywords. Limited to research articles. Limited to year 2008 to 2025. | 11 |
| EBSCO^2^ | Search words: nudg* AND cancer screening. Search within abstract. Limited to academic journals. Limited to year 2008 to 2025. | 26 |
| Total | | 101 |
| *Notes:*  ^1^Access to resources in databases is subject to Latvia's licensing agreements.  ^2^Articles from Academic Search Complete, Health Source: Nursing/Academic Edition, and MEDLINE. | | |

**Appendix C. Studies included in the literature review**

Studies are presented in chronological order.

| **Author,**  **(publication year)^1^** | **Title** | **Descriptive characteristics^2^** | **Nudge** | **Results^3^** |
| --- | --- | --- | --- | --- |
| Gupta et al., (2016) | Financial incentives for promoting colorectal cancer screening: A randomized, comparative effectiveness trial | **Study type:** Randomized, comparative effectiveness trial  **Measured effect:** Cancer screening uptake  **Country:** US  **Application context (cancer screening by localisation):** Colorectal  **(Perceived) nudge intervention performer:** Health care proffesionals  **Setting:** Conventional, digital  **Communication channel:** Mail, call  **Population (number of participants):** Uninsured adults aged 50–64 years, not up-to-date with colorectal cancer screening (8565 in final sample) | Mailed fecal immunochemical test (FIT) outreach, automated telephone reminders and financial microincentives ($5 or $10 gift card for returning the test kit. | FIT completion was 36.9% with vs. 36.2% without any financial incentive (P =0.60) and was also not statistically different for the $10 incentive (34.6%, P =0.32 vs. no incentive) or $5 incentive (39.2%, P =0.07 vs. no incentive) groups. |
| Schwartz et al., (2017) | Providing quantitative information and a nudge to undergo stool testing in a colorectal cancer screening decision aid: A randomized clinical trial | **Study type:** Randomized clinical trial  **Measured effect:** Cancer screening uptake (additionaly - patient-reported **screening intent,** perceived risk of cancer)  **Country:** US  **Application context (cancer screening by localisation):** Colorectal  **(Perceived) nudge intervention performer:** Study team members in healthcare facilities  **Setting:** Conventional  **Communication channel:** Direct in-person (with the decision aid provided in digital **format)**  **Population (number of participants):** Primary care patients aged 50–75 years who were eligible for colorectal cancer screening (213 in final sample) | Decision-aids in primary care sites including quantitative information and/or nudge towards stool testing providing encouraging messages with social context and addressing those who wish to procrastinate. | Patients who viewed the quantitative module had statistically significantly higher screening uptake (39%) than those who received only basic information (27%, p=0.012). In contrast, patients who viewed the nudge-based information, which included a social norm supporting stool testing, did not exhibit a statistically significant difference in screening uptake compared with the control group who received only basic information. |
| Stoffel et al., (2019) | Testing verbal quantifiers for social norms messages in cancer screening: Evidence from an online experiment | **Study type:** Online survay experiment  **Measured effect:** Intention to screen  **Country:** UK  **Application context (cancer screening by localisation):** Colorectal  **(Perceived) nudge intervention performer:** n/a  **Setting:** Digital  **Communication channel:** Online survey  **Population (number of participants):**  Respondents from a survey panel aged 35–54 who were eligible for colorectal cancer screening and not intending to do the test (1245 in final sample) | Verbal quantifiers in communication indicating social reference point of cancer screening uptake. | Verbal quantifiers increased screening intentions compared with the control group (from 7.8 to 12.5%, aOR 1.72; 95%CI 1.00–2.96 in the case of ‘a large number’ and 14.3%, aOR 2.02; 95% CI 1.20–3.38 for *‘near*ly half’), simply communicating that 43% do the test, however, had no impact on intentions (9.9% vs. 7.8% aOR 1.25; 95% CI 0.73–2.16). |
| Huf et al., (2020) | Behavioral economics informed message content in text message reminders to improve cervical screening participation: Two pragmatic randomized controlled trials | **Study type:** Pragmatic randomized controlled trials **Measured effect:** Cancer screening uptake  **Country:** UK  **Application context (cancer screening by localisation):** Cervical  **(Perceived) nudge intervention performer:** Health care proffesionals  **Setting:** Digital  **Communication channel:** SMS  **Population (number of participants):** In Study 1 (first invitation) women aged 24–29 (3139 randomised); in Study 2 (subsequent invitation) women aged aged 30–64 (11458 randomized) | SMS reminders to book screening that nudge by including social reference point (primary care practitioners name/ descriptive norm of attendance) or loss-framed or gain-framed messages. | In Study 1 participation was statistically significantly higher in the SMS-PCP arm (31.4%) compared to control (26.4%, aOR, 1.29, 95%CI: 1.09–1·51; p = 0.002). In Study 2 participation was highest in the SMS-PCP (38.4%) and SMS (38.1%) arms compared to control (34.4%), (aOR: 1.19, 95%CI: 1.03–1.38; p = 0.02 and aOR: 1.18, 95%CI: 1.02–1.37; p = 0.03, respectively). |
| Savicka & Circene, (2020) | Behaviour change interventions in breast and cervical cancer screening promotion | **Study type:** Intervention study  **Measured effect:** Cancer screening uptake  (additionally - increase of screenings performed in 6 general practitioner practices)  **Country: Latvia**  **Application context (cancer screening by localisation):** Breast and cervical  **(Perceived) nudge intervention performer:** Health care providers (call centre employees of general practitioner practices)  **Setting:** Conventional and digital  **Communication channel:** Call, SMS  **Population (number of participants):**  Women aged 50-69 eligible for breast cancer screening and women aged 25-70 eligible for cervical cancer screening (number of women not reported) | A direct offer by the healthcare provider call centre to perform breast and cervical cancer screening at a specific time without the need for a letter of invitation (those who did not pick up received SMS). | Prior to the intervention (2016), breast cancer screening uptake across general practitioner practices ranged from 20% to 47%, and cervical cancer screening uptake ranged from 9% to 28%. Following the intervention (2018), breast cancer screening uptake increased to a range of 57% to 100%, while cervical cancer screening uptake ranged from 59% to 106% (the rate exceeding 100% due to more screenings being completed than invitation letters sent). |
| Stoffel et al., (2020) | Offering male endoscopists as decoy option to nudge disinclined women to have colorectal cancer screening | **Study type:** Online survey experiments  **Measured effect:** Intention/choice to screen  **Country:** UK  **Application context (cancer screening by localisation):** Colorectal  **(Perceived) nudge intervention performer:** n/a  **Setting:** Digital  **Communication channel:** Online survey  **Population (number of participants):**  Women aged 35–54 from a survey panel who were eligible for colorectal cancer screening and not intending to do the test (in Experiment 1 (for intention outcomes) final sample 302; in Experiment 2 (for choice outcomes) final sample 300) | Examination appointment offerings for women including male practitioners (endoscopists) as decoy | While Experiment 1 showed that the presence of the decoy increased intentions to attend the appointment with the female practitioner (p = 0.02), Experiment 2 confirmed that women were more likely to choose the appointment with the female endoscopist if they were also offered the decoy (p<0.001). In the unadjusted logistic regression, women were more likely to choose the appointment with the female practitioner if it was offered together with the  appointment with the male practitioner (49.3% vs. 25.3%, OR 2.87, 95% CI: 1.76–4.67, p<0.001). This effect remained statistically significant after adjusting for covariates, including initial intention, sociodemographic variables, own perceived bowel cancer risk, cancer literacy score and numeracy (aOR) 2.62, 95% CI: 1.57–4.37, p<0.01). |
| Fukuyoshi et al., (2021) | Increasing hepatitis virus screening uptake at worksites in Japan using nudge theory and full subsidies | **Study type:** Cluster-randomised trial  **Measured effect:** Hepatitis virus screening uptake (additionaly - cost-effectiveness was calculated using the incremental cost-effectiveness ratio)  **Country:** Japan  **Application context (cancer screening by localisation):**  Liver (hepatitis virus screening – it itself is not labelled as cancer screening but it helps to identify people at higher risk of liver cancer, allowing for closer monitoring and potentially early detection of liver cancer)  **(Perceived) nudge intervention performer:** Health care proffesionals (during general checkups in workplace)  **Setting:** Conventional  **Communication channel:** Mail  **Population (number of participants):**  Employees of transportation company aged 35 to 74 years (1496) | Reminders to promote the hepatitis virus screening rates at worksites targeting employees with simplified messages and information about discounted or fully subsidized cost | The screening rate was 21.2%, 37.1%, and 86.3% for the control group (receiving simple reminder), group A (receiving nudge-based reminders with need for copayment) and group B (receiving nudge-based reminders with information about fully subsidized screening), respectively.  The risk ratio for group A was 1.75 (95% confidence interval [CI] 1.45–2.12) and that of group B was 4.08 (95% CI 3.44–4.83). The parameters of group A and group B also were significant when estimated using generalized linear mixed models. However, the cost-effectiveness (incremental cost-effectiveness ratio (ICER)) of the nudge-based reminder with the full subsidies was lower than that of only the nudge-based reminder. |
| Mizota & Yamamoto, (2021) | Rainbow of KIBOU project: Effectiveness of invitation materials for improving cancer screening rate using social marketing and behavioral economics approaches | **Study type:** Intervention study  **Measured effect:** Cancer screening uptake  **Country:** Japan  **Application context (cancer screening by localisation):**  Colorectal, breast, lung, cervical, stomach  **(Perceived) nudge intervention performer:** Municipalities (as health system providers)  **Setting:** Conventional  **Communication channel:** Mail  **Population (number of participants):**  residents (4.3 million from 787 municipalities) | Leaflets with colorectal, breast, lung, cervical, and stomach cancer screening recommendation materials including messages containing combination of nudges (additional and framed information, clear instructions, social reference) | Of 167 municipalities that were compared, 141 (83%) showed an increase in screening rate when materials were used. Overall, the screening rate improved by 2.6% or 1.44 fold (p < 0.001). |
| Potter et al., (2021) | Can targeting women with behavioural science 'nudges' help black men to find out more about their high risk of contracting prostate cancer? | **Study type:** Historically controlled intervention study  **Measured effect:** Click-through rate for provided information regarding cancer  **Country:** UK  **Application context (cancer screening by localisation):**  Prostate  **(Perceived) nudge intervention performer:** Health care proffesional  **Setting:** Digital  **Communication channel:** E-mail  **Population (number of participants):** wives and girl-friends of black men aged 45 and over and black men aged 45 and over (463 triallists, 315 women and 148 men) | Targeting black men or their wives/girlfriends via email with a picture of a black doctor suggesting prostate cancer screening for men, providing information about the risks, and including a link to find more information | 106 e-mail recipients (22.9%) clicked on the provided link. Notably, the click-through rate among men was 38.5%, substantially higher than that among women (15.5%). |
| Bucher et al., (2022) | Feasibility of a reinforcement learning-enabled digital health intervention to promote mammograms: Retrospective, single-arm, observational study | **Study type:** Retrospective, single-arm, observational study  **Measured effect:** Cancer screening uptake (scheduled and attended screenings)  **Country:** US  **Application context (cancer screening by localisation):**  Breast  **(Perceived) nudge intervention performer:** Health system provider  **Setting:** Digital  **Communication channel:** E-mail  **Population (number of participants):** Women aged 49.5 to 74 years who were eligible for breast cancer screening and suscribed to health system communications with a  valid email address (139 164 women received at least one intervention email) | Reinforcement learning–enabled emails incorporating nudges suggesting to attend mammography | 81.52% women engaged with at least one email. Deliverability of emails exceeded 98%. Among message recipients, 24.99% scheduled mammograms and 22.02% attended mammograms (88.08% attendance rate among women who scheduled appointments). |
| Orumaa et al., (2022) | Impact of the mobile game FightHPV on cervical cancer screening attendance: Retrospective cohort study | **Study type:** Retrospective cohort study  **Measured effect:** Cancer screening uptake  **Country:** Norway  **Application context (cancer screening by localisation):**  Cervical  **(Perceived) nudge intervention performer:** Public health authorities that developed the mobile app FightHPV / mobile app FightHPV itself  **Setting:** Digital  **Communication channel:** Mobile app  **Population (number of participants):** women aged 20 to 69 years who downloaded the app and were eligble for screening: (658 women in the intervention group and matched reference of 3860 women in control group) | Gamified educational cancer and its prevention related content via mobile app | 29.6% (195/658) of the women in the intervention group and 15.21% (587/3860) of those in the reference group underwent a cervical exam (P<.01). Women exposed to the FightHPV app were 2 times more likely to attend screening (adjusted HR 2.3,95% CI 2.0-2.7), during which they were 13 times more likely to be diagnosed with high-grade abnormality (adjusted HR 12.7, 95% CI 5.0-32.5) than the women in the reference group. |
| Stoffel et al., (2022) | Testing enhanced active choice to optimize acceptance and participation in a population-based colorectal cancer screening program in Malta | **Study type:** Randomized controlled trial  **Measured effect:** Cancer screening uptake  **Country:** Malta  **Application context (cancer screening by localisation):**  Colorectal  **(Perceived) nudge intervention performer:** Health system provider (via screening programm)  **Setting:** Conventional  **Communication channel:** Mail  **Population (number of participants):** Adults aged 60 to 64 who were eligible for colorectal cancer screening (8349) | Letters with information about colorectal cancer screening questioning interest in receiving a free fecal immunochemical test (FIT) test providing limited (yes/no) response options and framing of those responses | Overall, 48.4% (N=4042) accepted the invitation  and 42.4% (N=3542) did the screening test. While there were no statistically significant differences between the two conditions in terms of acceptance and participation, enhanced active choice did increase acceptance among men by 4.6 percentage points, which translated to a statistically significant increase in participation of 3.4 percentage points. |
| Gorini et al. (2023) | Testing behavioral economics messages to increase non-responders' participation in organized colorectal cancer-screening programs: A randomized controlled trial | **Study type:** Randomized controlled trial  **Measured effect:** Cancer screening uptake  **Country:** Italy  **Application context (cancer screening by localisation):**  Colorectal  **(Perceived) nudge intervention performer:** Health system provider (via screening programm)  **Setting:** Conventional  **Communication channel:** Mail  **Population (number of participants):** Non-responders of colorectal cancer-screening programs aged 54 to 70 years (11 505) | Invitation letters to screening providing normative feedback that invited person did not participate and/or social context that minority did not participate | Overall, screening participation rates were 5.3% in the control condition, 7.0% when providing normative feedback that invited person did not participate, 8.2% when providing social context that minority did not participate, and 7.4% when providing both normative feedback that invited person did not participate and social context that minority did not participate (*p* = 0.002). Invited subjects in the minority norm arm were more likely to participate (adjusted Odds Ratio[aOR] = 1.38; 95% Confidence Interval [95%CI,1.13–1.68]), particularly those aged 54–59 years (aOR = 1.52; 95%CI:1.16–1.98), and 60–64 (aOR = 1.57; 95% CI:1.62-; 95%CI: 1.06–2.48). Additionally, individuals aged 60–64 invited in feedback and feedback + minority norm arms demonstrated a higher likelihood of participation (aOR for feedback arm = 1.60; 95%CI: 1.06–2.41; aOR for feedback + minority norm arm = 1.99; 95%CI: 1.35–2.92). |
| Elfakki et al., (2024) | A nudge strategy to increase the uptake of colorectal cancer screening in Saudi Arabia: A pragmatic randomized trial in the Hail region | **Study type:** Pragmatic randomized trial  **Measured effect:** Cancer screening uptake  **Country:** Saudi Arabia  **Application context (cancer screening by localisation):**  Colorectal  **(Perceived) nudge intervention performer:** Health care proffesionals  **Setting:** Conventional, digital  **Communication channel:** Direct in-person, call, SMS  **Population (number of participants):** Adults aged 45 to 75 with an average colorectal cancer screening risk who used to receive their care and treatment at government primary care facilities (2061) | Direct positively framed health professional invitation to screening (family physicians and nurses in the intervention arm received on‑site nudging training: received recommendations regards the use of SMS and calls, and invitation language) | Increase in the proportion of the uptake of colorectal cancer screening is higher in the two intervention sites—Al Hait General Hospital Zone (38%) and Al‑Shamli General Hospital Zone (26%), than in the two control sites, Bagaa General Hospital Zone (18%) and King Khalid Hospital Zone (18%). |
| Maltz et al., (2024) | The framing effect of digital textual messages on uptake rates of medical checkups: Field study | **Study type:** Field study  **Measured effect:** Cancer screening uptake  **Country:** Israel  **Application context (cancer screening by localisation):**  Colorectal, breast, cervical (different health check-ups including these screenings)  **(Perceived) nudge intervention performer:** Health care proffesionals (health maintenance organization)  **Setting:** Digital  **Communication channel:** E-mail, SMS  **Population (number of participants):** Members of health maintenance organization (Maccabi Healthcare Services) aged 50 to 74 years (113 048) | Digital messages (emails/SMSs) using message framings on the uptake rates of medical check-ups (gain-framed, loss-framed, physician-recommended, linking to future, or emphasizing personal responsibility) | No statistically significant effect of message framing on uptake rates of medical checkups was observed. The rates of appointments for screening ranged from 12.9% to 14.1% across treatments. Based on a chi-square test, there was no evidence to reject the null hypothesis that these compliance rates are independent of the treatments (P=.35). |
| *Note.* ^1^Chronological order; when several studies published in the same year – alphabetical order. ^2^Study type stated as defined by authors. When indicating measured effect the measures that are aligned with the aims of this literature review are presented. ^3^Short extracts from study results are included for the purpose of review and analysis. Extracts are limited to key reported outcomes. Complete results and interpretation are available in the original publications. | | | | |

**Appendix D. Reported results and transformation to comparable effect expressed in pp changes**

Studies are presented in the same order as in Table 3.

| **Source** | **Results as reported in the original studies^1^** | **Transformation to comparable effect expressed in pp changes** |
| --- | --- | --- |
| Gorini et al. (2023) | Overall, screening participation rates were 5.3% in the control condition, 7.0% when providing normative feedback that invited person did not participate, 8.2% when providing social context that minority did not participate, and 7.4% when providing both normative feedback that invited person did not participate and social context that minority did not participate (*p* = 0.002). Invited subjects in the minority norm arm were more likely to participate (adjusted Odds Ratio[aOR] = 1.38; 95% Confidence Interval [95%CI,1.13–1.68]), particularly those aged 54–59 years (aOR = 1.52; 95%CI:1.16–1.98), and 60–64 (aOR = 1.57; 95% CI:1.62-; 95%CI: 1.06–2.48). Additionally, individuals aged 60–64 invited in feedback and feedback + minority norm arms demonstrated a higher likelihood of participation (aOR for feedback arm = 1.60; 95%CI: 1.06–2.41; aOR for feedback + minority norm arm = 1.99; 95%CI: 1.35–2.92). | Screening uptake differences between intervention groups and control group calculated:  1) with feedback of not participation:  7-5.3=1.7pp  2)with minority norm:  8.2-5.3=2.9pp  3) with both feedback and minority norm:  7.4-5.3=2.1pp  Conclusion:  *Increase in cancer screening uptake by 1,7pp with feedback of not participation, 2,9pp with minority norm, 2,1pp with both feedback of not participation and minority norm included compared to standard invitation letter.* |
| Huf et al., (2020) | In Study 1 participation was significantly higher in the SMS-PCP arm (31.4%) compared to control (26.4%, aOR, 1.29, 95%CI: 1.09–1·51; p = 0.002). In Study 2 participation was highest in the SMS-PCP (38.4%) and SMS (38.1%) arms compared to control (34.4%), (aOR: 1.19, 95%CI: 1.03–1.38; p = 0.02 and aOR: 1.18, 95%CI: 1.02–1.37; p = 0.03, respectively). | Screening uptake differences between intervention group and control group calculated:  31.4-26.4 = 5pp  38.4-34.4 = 4pp  Conclusion:  *Increase in cancer screening uptake by 4-5pp compared to no SMS. SMS reminders including name of primary care practitioners specifically yielded the largest improvements in uptake.* |
| Fukuyoshi et al., (2021) | The screening rate was 21.2%, 37.1%, and 86.3% for the control group (receiving simple reminder), group A (receiving nudge-based reminders with need for copayment) and group B (receiving nudge-based reminders with information about fully subsidized screening), respectively.  The risk ratio for group A was 1.75 (95% confidence interval [CI] 1.45–2.12) and that of group B was 4.08 (95% CI 3.44–4.83). The parameters of group A and group B also were significant when estimated using generalized linear mixed models. However, the cost-effectiveness (incremental cost-effectiveness ratio (ICER)) of the nudge-based reminder with the full subsidies was lower than that of only the nudge-based reminder. | Screening uptake differences between intervention groups and control group calculated:  1) with full subsidy  86.3-21.2=65.1pp  2)with only nudge-based reminder  37.1-21.2=15.9pp  Conclusion:  *Increase in cancer screening uptake with full subsidy 65.1pp and with only nudge-based reminder 15.9 pp compared to simple reminder. The cost-effectiveness higher for nudge-based reminder.* |
| Stoffel et al., (2019) | Verbal quantifiers increased screening intentions compared with the control group (from 7.8 to 12.5%, aOR 1.72; 95%CI 1.00–2.96 in the case of ‘a large number’ and 14.3%, aOR 2.02; 95% CI 1.20–3.38 for *‘near*ly half’), simply communicating that 43% do the test, however, had no impact on intentions (9.9% vs. 7.8% aOR 1.25; 95% CI 0.73–2.16). | Screening intention differences between intervention groups and control group calculated:  1)with formulation ‘a large number’  12.5-7.8=4.7pp  2)with formulation ‘nearly half’  14.3-7.8=6.5pp  Conclusion:  *Increase in cancer screening intentions by 4.7pp when formulation “a large number” was used and by 6.5pp when formulation “nearly half” was used compared to control group that did not contain any information on uptake.* |
| Stoffel et al., (2020) | While Experiment 1 showed that the presence of the decoy increased intentions to attend the appointment with the female practitioner (p = 0.02), Experiment 2 confirmed that women were more likely to choose the appointment with the female endoscopist if they were also offered the decoy (p<0.001). In the unadjusted logistic regression, women were more likely to choose the appointment with the female practitioner if it was offered together with the  appointment with the male practitioner (49.3% vs. 25.3%, OR 2.87, 95% CI: 1.76–4.67, p<0.001). This effect remained statistically significant after adjusting for covariates, including initial intention, sociodemographic variables, own perceived bowel cancer risk, cancer literacy score and numeracy (aOR) 2.62, 95% CI: 1.57–4.37, p<0.01). | Screening choice differences between intervention groups and control group calculated:  49.3-25.3=24pp.  Conclusion:  *Increased intention and choice to be screened with female practitioner in the presence of the decoy of men practitioner by 24pp compared to an appointment with a female practitioner.* |
| Gupta et al., (2016) | FIT completion was 36.9% with vs. 36.2% without any financial incentive (P =0.60) and was also not statistically different for the $10 incentive (34.6%, P =0.32 vs. no incentive) or $5 incentive (39.2%, P =0.07 vs. no incentive) groups. | Conclusion:  *Cancer screening uptake not significantly influenced by financial microincentives compared to simply provision of mailed test.*  *FIT completion 36.2% with FIT outreach without any financial incentive. (Due to the lack of control group without FIT outreach, standardization to pp increases not possible.)* |
| Schwartz et al., (2017) | Patients viewing the quantitative module were more likely to be screened than those who did not (p=0.012). Patients viewing the nudge module had a greater increase in perceived colorectal cancer risk than those who did not (p=.041). Those viewing the quantitative module had a smaller increase in perceived risk than those who did not (p=.046), and the effect was moderated by numeracy. Among patients with high numeracy who did not view the nudge module, those who viewed the quantitative module had a greater increase in intent to undergo FIT (p=.028) than did those who did not.  According to Table 5 (“CRC Uptake by Group and Numeracy Level”) in the original study, CRC screening uptake data are as follows:  - Basic Info Only n/N (%) = 14/52 (27%)  - Quantitative n/N (%) = 22/56 (39%)  - Final Model p-value = 0.013 | Screening choice differences between quantitative information receivers and basic info receivers as control group:  39-27=12pp  Conclusion:  *Increase in cancer screening uptake for patients that viewed the quantitative information by 12pp compared to those who had basic info, while nudge-based information does not provide significant effect.* |
| Stoffel et al., (2022) | Overall, 48.4% (N=4042) accepted the invitation and 42.4% (N=3542) did the screening test. While there were no statistically significant differences between the two conditions in terms of acceptance and participation, enhanced active choice did increase acceptance among men by 4.6 percentage points, which translated to a significant increase in participation of 3.4 percentage points. | Conclusion:  *Increase in cancer screening uptake by enhanced active choice among men by 3.4pp compared to standard invitation with opt-in strategy, but no significant effect among women.* |
| Maltz et al., (2024) | No significant effect of message framing on uptake rates of medical checkups was observed. The rates of appointments for screening ranged from 12.9% to 14.1% across treatments. Based on a chi-square test, there was no evidence to reject the null hypothesis that these compliance rates are independent of the treatments (P=.35). | Conclusion:  *No significant effect of message framing on uptake rates of medical check-ups compared to neutrally framed messages.* |
| Bucher et al., (2022) | 81.52% women engaged with at least one email. Deliverability of emails exceeded 98%. Among message recipients, 24.99% scheduled mammograms and 22.02% attended mammograms (88.08% attendance rate among women who scheduled appointments). | Conclusion:  *Cancer screening uptake of 24.99% scheduled and 22.02% attended to screening of patients who were overdue. (Due to the lack of control group, standardization to pp increases not possible.)* |
| Elfakki et al., (2024) | Increase in the proportion of the uptake of colorectal cancer screening is higher in the two intervention sites—Al Hait General Hospital Zone (38%) and Al‑Shamli General Hospital Zone (26%), than in the two control sites, Bagaa General Hospital Zone (18%) and King Khalid Hospital Zone (18%). | Screening uptake differences between intervention sites and control sites calculated:  38-18 = 20pp  26-18=8pp  Conclusion:  *Increase in cancer screening uptake by 8-20pp compared to routine care.* |
| Mizota & Yamamoto, (2021) | From Of 167 municipalities that were compared, 141 (83%) showed an increase in screening rate when materials were used. Overall, the screening rate improved by 2.6% or 1.44 fold (p < 0.001).  According to Table 5 (“Comparison of cancer screening rates between group using the ROK* materials and control”) in the original study, participation rate difference (ROK-control) (%) are as follows:  - Colorectum: 1.7%  - Breast: 3.0%  - Lung: 2.1%  - Cervix: 3.5%  - Stomach: 2.2%  p<0,001  *ROK: Rainbow of KIBOU project. | Conclusion:  *Increase in cancer screening uptake by 2.6pp compared to historical control (by 1.7pp for colorectal, by 3,0pp for breast, by 2.1pp for lung, by 3.5pp for cervical, by 2.2pp for stomach cancer screening).* |
| Orumaa et al., (2022) | 29.6% (195/658) of the women in the intervention group and 15.21% (587/3860) of those in the reference group underwent a cervical exam (P<.01). Women exposed to the FightHPV app were 2 times more likely to attend screening (adjusted HR 2.3,95% CI 2.0-2.7), during which they were 13 times more likely to be diagnosed with high-grade abnormality (adjusted HR 12.7, 95% CI 5.0-32.5) than the women in the reference group. | Screening uptake differences between intervention group and reference group calculated:  29.6-15.21=14.39pp  Conclusion:  *Increase in cancer screening uptake by 14.4pp for app users 6 months after enrolment compared to comparable historical control group not exposed to app.* |
| Potter et al., (2021) | In total 463 emails were sent. 106 e-mail recipients (22.9%) clicked on the provided link. Notably, the click-through rate among men was 38.5%, substantially higher than that among women (15.5%).  Standard Prostate Cancer UK email had a click-through rate of 6.3%. | The click-through rate differences calculated:  1) for men:  38.5-6.3=32.2pp  2) for women:  15.5-6.3=9.2pp  Conclusion:  *Increased click-through rate for provided information regarding cancer for man by 32.2pp and for woman by 9.2pp compared to Prostate Cancer UK click-through rate.* |
| Savicka & Circene, (2020) | Prior to the intervention (2016), breast cancer screening uptake across general practitioner practices ranged from 20% to 47%, and cervical cancer screening uptake ranged from 9% to 28%. Following the intervention (2018), breast cancer screening uptake increased to a range of 57% to 100%, while cervical cancer screening uptake ranged from 59% to 106% (the rate exceeding 100% due to more screenings being completed than invitation letters sent).  According to Table 4 (“Results or performed screenings in GP practices before the intervention study (2014-2016) and during the pilot intervention study (2017) and intervention study (2018)”) cancer screening uptake data are as follows:  1) for breast cancer screening:  - Number of screenings performed in 2018:  General Practitioner Nr.1: 31  General Practitioner Nr.2: 7  General Practitioner Nr.3: 29  General Practitioner Nr.4: 24  General Practitioner Nr.5: 17  General Practitioner Nr.6: 18  - Sent letters in 2018:  General Practitioner Nr.1: 31  General Practitioner Nr.2: 7  General Practitioner Nr.3: 51  General Practitioner Nr.4: 34  General Practitioner Nr.5: 25  General Practitioner Nr.6: 27  - Number of screenings performed in 2016:  General Practitioner Nr.1: 46  General Practitioner Nr.2: 22  General Practitioner Nr.3: 48  General Practitioner Nr.4: 51  General Practitioner Nr.5: 21  General Practitioner Nr.6: 38  - Sent letters in 2016:  General Practitioner Nr.1: 101  General Practitioner Nr.2: 112  General Practitioner Nr.3: 184  General Practitioner Nr.4: 113  General Practitioner Nr.5: 83  General Practitioner Nr.6: 81  2) for cervical cancer screening:  Number of screenings performed in 2018:  General Practitioner Nr.1: 45  General Practitioner Nr.2: 11  General Practitioner Nr.3: 41  General Practitioner Nr.4: 53  General Practitioner Nr.5: 21  General Practitioner Nr.6: 51  - Sent letters in 2018:  General Practitioner Nr.1: 59  General Practitioner Nr.2: 11  General Practitioner Nr.3: 70  General Practitioner Nr.4: 63  General Practitioner Nr.5: 33  General Practitioner Nr.6: 48  - Number of screenings performed in 2016:  General Practitioner Nr.1: 54  General Practitioner Nr.2: 10  General Practitioner Nr.3: 51  General Practitioner Nr.4: 55  General Practitioner Nr.5: 28  General Practitioner Nr.6: 44  - Sent letters in 2016:  General Practitioner Nr.1: 193  General Practitioner Nr.2: 115  General Practitioner Nr.3: 184  General Practitioner Nr.4: 199  General Practitioner Nr.5: 153  General Practitioner Nr.6: 157 | Screening uptake differences before and after intervention calculated jointly for all GP practices:  1) for breast cancer screening:  - uptake in 2018: (31+7+29+24+17+18)/(31+7+51+34+25+27)= 126/175=72%  - uptake in 2016:  (46+22+48+51+21+38)/(101+112+184+113+83+81)= 226/674=34%  - increase:  72-34=38pp  2)for cervical cancer screening:  - uptake in 2018:  (45+11+41+53+21+51)/(59+11+70+63+33+48)=  222/284=78%  - uptake in 2016:  (54+10+51+55+28+44)/(193+115+184+199+153+157)=  242/1001=24%  - increase:  78-24=54pp  Conclusion:  *Increase in cancer screening uptake by 38pp for breast cancer and by 54pp for cervical cancer screening (post intervention year 2018 compared to pre-intervention year 2016).* |
| *Note.* ^1^Short extracts from study results are included solely for the purpose of review and comparative analysis across studies. Extracts are limited to key reported outcomes and used to derive comparable percentage point (pp) changes. Complete results and interpretation are available in the original publications. | | |

**Appendix E**. Risk of bias assessment – an overview

| **Author (year)** | **Methodological quality criteria** | | | | | | | | | | | | | | | | | **Total score out of 7**  (1 point for each “Yes”) | **Risk of bias**^4^  (≥ 5 points – acceptable;  <5 points – high risk of bias) |
| --- | --- | --- | --- | --- | --- | --- | --- | --- | --- | --- | --- | --- | --- | --- | --- | --- | --- | --- | --- |
|  | **Screening** | | **Quantitative randomized controlled trials**^1^ | | | | | **Quantitative non-randomized** | | | | | **Quantitative descriptive** | | | | |  |  |
|  | Are there clear research questions? | Do the collected data allow to address the research questions? | Is randomization appropriately performed? | Are the groups comparable at baseline? | Are there complete outcome data?^2^ | Are outcome assessors blinded to the intervention provided?^3^ | Did the participants adhere to the assigned intervention? | Are the participants representative of the target population? | Are measurements appropriate regarding both the outcome and intervention (or exposure)? | Are there complete outcome data?^2^ | Are the confounders accounted for in the design and analysis? | During the study period, is the intervention administered (or exposure occurred) as intended? | Is the sampling strategy relevant to address the research question? | Is the sample representative of the target population? | Are the measurements appropriate? | Is the risk of nonresponse bias low? | Is the statistical analysis appropriate to answer the research question? |  |  |
| Bucher et al., 2022 | Yes | Yes |  |  |  |  |  |  |  |  |  |  | Yes | Yes | Yes | Can’t tell | Yes | 6 | Acceptable |
| Elfakki et al., 2024 | Yes | Yes | No | Can’t tell | No | No | Yes |  |  |  |  |  |  |  |  |  |  | 3 | High risk |
| Fukuyoshi et al., 2021 | Yes | Yes | Yes | Yes | Yes | Can’t tell | Yes |  |  |  |  |  |  |  |  |  |  | 6 | Acceptable |
| Gorini et al., 2023 | Yes | Yes | Yes | Yes | Yes | No | Yes |  |  |  |  |  |  |  |  |  |  | 6 | Acceptable |
| Gupta et al., 2016 | Yes | Yes | Yes | Yes | Yes | No | Yes |  |  |  |  |  |  |  |  |  |  | 6 | Acceptable |
| Huf et al., 2020 | Yes | Yes | Yes | Yes | Yes | No | Yes |  |  |  |  |  |  |  |  |  |  | 6 | Acceptable |
| Maltz et al., 2024 | Yes | Yes | Yes | Yes | Yes | No | Yes |  |  |  |  |  |  |  |  |  |  | 6 | Acceptable |
| Mizota & Yamamoto, 2021 | Yes | Yes |  |  |  |  |  | Yes | Can’t tell | Yes | No | Yes |  |  |  |  |  | 5 | Acceptable |
| Orumaa et al., 2022 | Yes | Yes |  |  |  |  |  | Yes | Yes | Yes | Yes | Yes |  |  |  |  |  | 7 | Acceptable |
| Potter et al., 2021 | Yes | Yes |  |  |  |  |  | Can’t tell | Yes | No | No | Can’t tell |  |  |  |  |  | 3 | High risk |
| Savicka & Circene, 2020 | Yes | Yes |  |  |  |  |  |  |  |  |  |  | Can’t tell | No | No | Can’t tell | No | 2 | High risk |
| Schwartz et al., 2017 | Yes | Yes | Yes | Yes | Yes | No | Yes |  |  |  |  |  |  |  |  |  |  | 6 | Acceptable |
| Stoffel et al., 2019 | Yes | Yes | Yes | Yes | Yes | No | Yes |  |  |  |  |  |  |  |  |  |  | 6 | Acceptable |
| Stoffel et al., 2020 | Yes | Yes | Yes | Yes | Yes | No | Yes |  |  |  |  |  |  |  |  |  |  | 6 | Acceptable |
| Stoffel et al., 2022 | Yes | Yes | Yes | Can’t tell | Yes | No | Yes |  |  |  |  |  |  |  |  |  |  | 5 | Acceptable |
| Notes: ^1^Although not field-based, survey experiments with random allocation and multiple researcher-assigned conditions (Stoffel et al., 2019; Stoffel et al., 2020) according to MMAT were classified as randomized controlled trials. ^2^The MMAT methodological quality criterion “Are there complete outcome data?” was interpreted to encompass both the completeness of outcome data and whether the data were statistically tested and appropriately analyzed to allow group comparisons. ^3^Outcome assessor blinding is often not feasible in behavioral intervention studies because the nature of the intervention is visible, often involves subjective self-reported outcomes, and assessors may also be involved in delivering the intervention. Although risk of bias is acknowledged, this does not substantially reduce the overall quality of evidence when outcomes are objectively measured or consistently assessed across groups. ^4^Quantitative randomized controlled trials with an acceptable risk of bias are marked in green, representing studies that provide the highest quality evidence for evaluating the effectiveness of nudging. | | | | | | | | | | | | | | | | | | | |

**Appendix F**. Risk of bias assessment – detailed

| **Author (year)** | **MMAT study category** | **Study category justification** | **MMAT methodological quality criteria** | **Responses** | | | |
| --- | --- | --- | --- | --- | --- | --- | --- |
|  |  |  |  | **Yes** | **No** | **Can’t tell** | **Comments** |
| Bucher et al., 2022 | Quantitative - descriptive | The study reports data from a single group exposed to a digital health intervention, without the inclusion of a control or comparison group.  Therefore, it is a non-comparative quantitative study and is classified as a quantitative descriptive study. | S1. Are there clear research questions? | X |  |  | The study clearly aims to assess the feasibility of a digital, AI-enabled email intervention to promote mammogram uptake. |
|  |  |  | S2. Do the collected data allow to address the research questions? | X |  |  | Data on email engagement (opens, clicks), behavioral outcomes (scheduled/attended mammograms), and demographics were collected and analyzed. |
|  |  |  | 4.1. Is the sampling strategy relevant to address the research question? | X |  |  | Targets overdue patients. |
|  |  |  | 4.2. Is the sample representative of the target population? | X |  |  | Large health system patient base used. Over 139 000 women from the target population with diverse demographics, and subgroup analyses showed proportional engagement across age, race, income, and education levels. |
|  |  |  | 4.3. Are the measurements appropriate? | X |  |  | Open rate, scheduling, attendance are appropriate behavioral metrics. |
|  |  |  | 4.4. Is the risk of nonresponse bias low? |  |  | X | 10.5% unsubscribed from the intervention messaging and no analysis was provided on characteristics of nonresponders. Therefore, risk of nonresponse bias cannot be fully assessed. |
|  |  |  | 4.5. Is the statistical analysis appropriate? | X |  |  | Descriptive + chi-square tests used. |
| Elfakki et al., 2024 | Quantitative - randomized controlled trial | The study employed cluster randomization to assign participants to either an intervention or control group, aiming to evaluate the effectiveness of a nudge-based strategy on colorectal cancer screening uptake. The intervention consisted of nudging techniques delivered by family physicians and nurses, while the control group received usual care without any behavioral prompts.  The study meets the criteria of a randomized controlled trial. | S1. Are there clear research questions? | X |  |  | The study clearly aims to examine the effectiveness of using nudging in primary healthcare settings to encourage patients to participate in colorectal cancer screening. |
|  |  |  | S2. Do the collected data allow to address the research questions? | X |  |  | Data on uptake rates and demographics were collected and analyzed. |
|  |  |  | 2.1. Is randomization appropriately performed? |  | X |  | Although cluster randomization is reported, no details are provided on cluster generation, and only four clusters were used, which increases the risk of bias. |
|  |  |  | 2.2. Are the groups comparable at baseline? |  |  | X | No detailed baseline characteristics or statistical comparisons were reported. |
|  |  |  | 2.3. Are there complete outcome data? |  | X |  | Screening uptake data were fully reported across all sites, including a flowchart and sample sizes. However, no data were provided regarding statistical testing to compare the effectiveness between groups, which limits the analysis of the intervention’s impact. |
|  |  |  | 2.4. Are outcome assessors blinded to the intervention? |  | X |  | Open-label trial; blinding not possible or reported. |
|  |  |  | 2.5. Did participants adhere to the assigned intervention? | X |  |  | Intervention delivery through trained physicians and nurses and uptake were clearly described; training and engagement strategies were reported. No crossovers or reported deviations were reported. |
| Fukuyoshi et al., 2021 | Quantitative - randomized controlled trial | The study compares three intervention arms: a control group receiving only a standard reminder, a group receiving a nudged reminder with co-payment, and a group receiving a nudged reminder with full financial subsidy. Participants were randomly assigned through a cluster-randomized design, with thirteen worksites (tracking stations) allocated to one of the three groups. The intervention was designed and implemented by researchers to assess its impact on hepatitis screening uptake. Statistical tests were applied to analyse group differences. The study meets the criteria of a randomized controlled trial. | S1. Are there clear research questions? | X |  |  | The study clearly aims to examine the effectiveness of using nudging or subsidies to increase hepatitis screening uptake, with the goal of reducing liver cancer mortality. |
|  |  |  | S2. Do the collected data allow to address the research questions? | X |  |  | Data on screening uptake, timing of attendance, and demographics were collected and analyzed. |
|  |  |  | 2.1. Is randomization appropriately performed? | X |  |  | Random assignment of 13 clusters to 3 arms is clearly stated, and group comparability is assessed. |
|  |  |  | 2.2. Are the groups comparable at baseline? | X |  |  | No significant differences in age and gender between groups. |
|  |  |  | 2.3. Are there complete outcome data? | X |  |  | Screening outcomes were reported for all participants who met inclusion criteria; no missing data indicated. Statistical testing was performed. Data for comparing the effectiveness between groups were provided. |
|  |  |  | 2.4. Are outcome assessors blinded to the intervention? |  |  | X | The study does not mention blinding. Given the intervention design (visible group differences and workplace-level implementation), it is likely that those recording screening attendance were aware of the group assignments. |
|  |  |  | 2.5. Did participants adhere to the assigned intervention? | X |  |  | Intervention delivery followed plan per cluster. No crossovers or reported deviations were reported. |
| Gorini et al., 2023 | Quantitative - randomized controlled trial | The study assessed the impact of behaviourally framed letters on screening participation by comparing a standard control letter with three message variants. Participants were individually randomized into four groups using a computer-generated sequence. The interventions—invitation letters incorporating different behavioural economic messages—were assigned by researchers and applied at the individual level. Statistical comparisons were conducted across the randomized arms to evaluate differences in outcomes. The study meets the criteria of a randomized controlled trial. | S1. Are there clear research questions? | X |  |  | The study clearly aims to evaluate whether adding behavioral economics-inspired messages to invitation letters increases colorectal cancer screening participation among previous non-responders. |
|  |  |  | S2. Do the collected data allow to address the research questions? | X |  |  | Data on colorectal cancer screening participation and demographics were collected and analyzed. |
|  |  |  | 2.1. Is randomization appropriately performed? | X |  |  | Participants were randomized using a computer-based pseudo-random number generator. |
|  |  |  | 2.2. Are the groups comparable at baseline? | X |  |  | Baseline characteristics (age, gender, place of birth) were compared and reported. Groups were generally comparable after adjusting for age and gender. |
|  |  |  | 2.3. Are there complete outcome data? | X |  |  | Screening outcomes were reported for all included participants; no missing data indicated. Statistical testing was performed. Data for comparing the effectiveness between groups were provided. |
|  |  |  | 2.4. Are outcome assessors blinded to the intervention? |  | X |  | The study explicitly states that neither researchers nor participants were blinded. |
|  |  |  | 2.5. Did participants adhere to the assigned intervention? | X |  |  | Each group received the assigned invitation letter. No cross-over or deviations were reported. |
| Gupta et al., 2016 | Quantitative - randomized controlled trial | The study compared the effects of different financial incentives on screening uptake by randomly assigning uninsured adults to one of three groups: mailed outreach only, outreach with a $5 incentive or outreach with a $10 incentive. The interventions were implemented at the individual level, and the study followed a comparative effectiveness trial design, incorporating random allocation and outcome evaluation through statistical analysis. The study meets the criteria of a randomized controlled trial. | S1. Are there clear research questions? | X |  |  | The study clearly aims to investigates whether small financial incentives ($5 or $10) improve completion of mailed fecal immunochemical test (FIT) screening. |
|  |  |  | S2. Do the collected data allow to address the research questions? | X |  |  | Data on FIT return rates and demographics were collected and analyzed. |
|  |  |  | 2.1. Is randomization appropriately performed? | X |  |  | A computer-generated randomization process was used. |
|  |  |  | 2.2. Are the groups comparable at baseline? | X |  |  | Baseline characteristics (age, sex, race/ethnicity, neighborhood poverty) were found to be similar across groups. |
|  |  |  | 2.3. Are there complete outcome data? | X |  |  | FIT outcomes were reported for all randomized participants; no missing data indicated. Statistical testing was performed. Data for comparing the effectiveness between groups were provided. |
|  |  |  | 2.4. Are outcome assessors blinded to the intervention? |  | X |  | The study does not mention blinding. Likely not feasible due to intervention visibility (e.g., incentive message included in letters). |
|  |  |  | 2.5. Did participants adhere to the assigned intervention? | X |  |  | Interventions were implemented as planned. No cross-over or deviations were reported. |
| Huf et al., 2020 | Quantitative- randomized controlled trial | The study evaluated the effectiveness of behaviorally framed SMS reminders on cervical screening uptake through two parallel randomized trials. Participants were randomly assigned to receive one of several message types (e.g., gain framing, loss framing, social norms) or no message (control). Comparisons of screening outcomes were conducted across all study arms.  The study meets the criteria of a randomized controlled trial. | S1. Are there clear research questions? | X |  |  | The study clearly aims to evaluate if SMS reminders with various message framings improve cervical screening participation. |
|  |  |  | S2. Do the collected data allow to address the research questions? | X |  |  | Screening outcomes and participant demographics were collected and analyzed. |
|  |  |  | 2.1. Is randomization appropriately performed? | X |  |  | Randomization by pseudorandom number generator is described for both trials. |
|  |  |  | 2.2. Are the groups comparable at baseline? | X |  |  | Mean age and deprivation index were similar across arms; no substantial imbalance noted. |
|  |  |  | 2.3. Are there complete outcome data? | X |  |  | Screening outcomes were reported for all analyzed participants with flow diagrams included. Large exclusions were due to predefined eligibility filters and system constraints, not due to dropout during follow-up. Statistical testing was performed. Data for comparing the effectiveness between groups were provided. |
|  |  |  | 2.4. Are outcome assessors blinded to the intervention? |  | X |  | Open-label design; blinding not possible or reported (SMS content could not be blinded to participants or researchers). |
|  |  |  | 2.5. Did participants adhere to the assigned intervention? | X |  |  | SMS interventions were delivered as assigned. No cross-over or deviations were reported. |
| Maltz et al., 2024 | Quantitative – randomized controlled trial | This large-scale digital field experiment randomly assigned individuals to one of six message framings (gain, loss, recommendation, implementation, empowerment, or control) aimed at promoting preventive health behaviors. All exposures were assigned by the researchers, and statistical comparisons were conducted across groups. The study meets the criteria of a randomized controlled trial. | S1. Are there clear research questions? | X |  |  | The study clearly aims to assess the impact of behavioral framing on uptake of medical checkups. |
|  |  |  | S2. Do the collected data allow to address the research questions? | X |  |  | Screening appointment scheduling, test completion and demographics were collected and analyzed. |
|  |  |  | 2.1. Is randomization appropriately performed? | X |  |  | Random assignment into groups is stated. Group balance confirmed. |
|  |  |  | 2.2. Are the groups comparable at baseline? | X |  |  | Groups were well balanced on observable characteristics (age, gender, socioeconomic status, medical conditions). |
|  |  |  | 2.3. Are there complete outcome data? | X |  |  | Screening outcomes were reported for all included participants; no missing data indicated. Statistical testing was performed. Data for comparing the effectiveness between groups were provided. |
|  |  |  | 2.4. Are outcome assessors blinded to the intervention? |  | X |  | Open-label design; intervention content (messages) was visible to both participants and researchers. |
|  |  |  | 2.5. Did participants adhere to the assigned intervention? | X |  |  | Participants received messages as assigned. No cross-over or deviations were reported. |
| Mizota & Yamamoto, 2021 | Quantitative non-randomized | Study uses a bfore–after comparison of municipalities that voluntarily adopted standardized invitation materials. There was no random allocation of participants to intervention or control groups. The design relies on observational, real-world implementation across municipalities, using historical controls, making this a  quantitative non-randomized study. | S1. Are there clear research questions? | X |  |  | The study clearly aims to assess the real-world effectiveness of behavioral economics and social marketing-based cancer screening invitation materials. |
|  |  |  | S2. Do the collected data allow to address the research questions? | X |  |  | Uptake rates, number of invitations, and comparisons to historical control rates were collected and analyzed. |
|  |  |  | 3.1. Are the participants representative of the target population? | X |  |  | The study included 4.3 million residents from 787 municipalities across Japan, representing diverse settings and demographics. |
|  |  |  | 3.2. Are measurements appropriate regarding both the outcome and intervention (or exposure)? |  |  | X | Screening participation was objectively and appropriately measured through administrative data. The intervention (distribution of behaviorally informed materials) was described, but variation in implementation across municipalities and limited detail on actual delivery and exposure reduce certainty about how uniformly the intervention was applied. |
|  |  |  | 3.3. Are there complete outcome data? | X |  |  | Screening rates were reported in detail for all available participants, across multiple cancer types and years.  Statistical testing was performed. Data for comparing the effectiveness with historical controls were provided. |
|  |  |  | 3.4. Are the confounders accounted for in the design and analysis? |  | X |  | The study did not adjust for potential confounders - comparisons were made with historical data without controlling for differences between municipalities. |
|  |  |  | 3.5. During the study period, is the intervention administered (or exposure occurred) as intended? | X |  |  | The intervention was implemented at the municipal level as planned. Using the RE-AIM (Reach, Effectiveness, Adaption, Implementation, Maintenance) framework, the study documented adoption rates and materials used, confirming that the intervention was delivered as intended, despite some variation across municipalities. |
| Orumaa et al., 2022 | Quantitative non-randomized | The study compares outcomes between women exposed and not exposed to a gamified mobile application (FightHPV). Researchers observed results following natural (non-assigned) exposure to the app.  It employed a matched retrospective cohort design, where participants were not randomly assigned to intervention or control groups. Instead, matching was conducted post hoc based on age and screening history. Therefore, the study is best classified as a quantitative non-randomized study. | S1. Are there clear research questions? | X |  |  | The study clearly aims to examine the association between exposure to the FightHPV mobile app and having a cervical exam. |
|  |  |  | S2. Do the collected data allow to address the research questions? | X |  |  | Screening attendance and outcomes were collected and analyzed. |
|  |  |  | 3.1. Are the participants representative of the target population? | X |  |  | The study population consisted of Norwegian women selected from the national cancer screening registry - aged 20–69 years, which matches the target population for cervical cancer screening in Norway. |
|  |  |  | 3.2. Are measurements appropriate regarding both the outcome and intervention (or exposure)? | X |  |  | Cervical screening participation was measured using reliable national registry data. App exposure was clearly defined by user registration and consent. |
|  |  |  | 3.3. Are there complete outcome data? | X |  |  | No mention of missing outcome data; all results were retrieved from comprehensive national registries. Statistical testing was performed. Data for comparing the effectiveness between groups were provided. |
|  |  |  | 3.4. Are the confounders accounted for in the design and analysis? | X |  |  | The study accounted for confounders through matching on age and screening history, and adjusted for education, employment, marital status, income, and country of birth in the analysis. |
|  |  |  | 3.5. During the study period, is the intervention administered (or exposure occurred) as intended? | X |  |  | Exposure was defined by consent to use the app. The intervention (app exposure) was not interfered with or modified during the follow-up. |
| Potter et al., 2021 | Quantitative - non-randomized | The study evaluates click-through rates in response to a social norm nudge. Although the authors refer to it as a “trial,” it does not meet the criteria for a true randomized controlled trial. Participants were not randomly assigned; instead, the study relied on historical controls and comparisons across self-selected organizations, representing a non-random sample. Therefore, it is best classified as a quantitative non-randomized study. | S1. Are there clear research questions? | X |  |  | The study clearly aims to examine whether targeting women using behavioral nudges can influence black men’s prostate cancer awareness. |
|  |  |  | S2. Do the collected data allow to address the research questions? | X |  |  | Click-through rates as a proxy for engagement, separated by gender and compared to historical controls. |
|  |  |  | 3.1. Are the participants representative of the target population? |  |  | X | Participants were drawn from Afro-Caribbean organizations and selected by the organizers; this is a convenience sample and may not represent the broader population of black men and women in the UK. |
|  |  |  | 3.2. Are measurements appropriate regarding both the outcome and intervention (or exposure)? | X |  |  | Click-through rates are a valid behavioral proxy for engagement; the intervention (email content and delivery method) is appropriate and clearly described. |
|  |  |  | 3.3. Are there complete outcome data? |  | X |  | Outcomes (clicks) were recorded via Google Analytics for all emails sent, with no mention of missing data. Although statistical testing was conducted—specifically a chi-square test examining differences in click-through rates between male and female recipients—no statistical comparison was presented to directly evaluate the effectiveness of the intervention compared to the control or historical baseline. This limits the limits the analysis of the intervention’s impact. |
|  |  |  | 3.4. Are the confounders accounted for in the design and analysis? |  | X |  | The study does not account for potential confounders such as prior awareness, prior exposure to prostate cancer messaging, etc. |
|  |  |  | 3.5. During the study period, is the intervention administered (or exposure occurred) as intended? |  |  | X | Although instructions were given to organizations, variability in email delivery and potential sharing/discussion among recipients is acknowledged as a limitation. Implementation fidelity is uncertain. |
| Savicka & Circene, 2020 | Quantitative - descriptive | This is an intervention study using a pre–post design without a control group, comparison arms, or randomization. The study evaluates the effect of a tailored communication intervention on screening attendance by comparing rates before and after implementation across six GP practices. As the data are primarily descriptive (e.g., percent change in attendance), the study is best classified as a quantitative descriptive study. | S1. Are there clear research questions? | X |  |  | The study clearly aims to assess the effect of behavioral economics-based communication (nudge + midwife contact) on increasing cervical and breast cancer screening uptake. |
|  |  |  | S2. Do the collected data allow to address the research questions? | X |  |  | Screening participation rates before and after the intervention were collected and compared across six GP practices. |
|  |  |  | 4.1. Is the sampling strategy relevant to address the research question? |  |  | X | While the sample seems relevant in principle (non-attending women from GP practices), the sample included women from only 6 GP practices, the sampling method is not fully described, and no justification is given for site selection. |
|  |  |  | 4.2. Is the sample representative of the target population? |  | X |  | The sample is geographically limited to patients of six GP practices in one location, which may not reflect the broader screening-eligible population in Latvia. |
|  |  |  | 4.3. Are the measurements appropriate? |  | X |  | Screening participation was measured using clinic records, with reported pre/post numbers of examinations, but there is lack of details about how the data were collected or verified**.** Outcome measurements for practices were not aggregated, which hindered a clear and coherent evaluation of the intervention’s overall effectiveness. |
|  |  |  | 4.4. Is the risk of nonresponse bias low? |  |  | X | No information is provided on how many women refused or did not respond, nor are their characteristics analyzed. Therefore, risk of nonresponse bias is high or unclear. |
|  |  |  | 4.5. Is the statistical analysis appropriate? |  | X |  | Only percent increases are reported. No statistical significance testing or confidence intervals are provided to assess the strength of the observed effects. |
| Schwartz et al., 2017 | Quantitative - randomized controlled trial | In this study, participants were randomly assigned to one of four decision aid formats: basic information only, quantitative information, a nudge to stool testing, or a combination of quantitative information and a nudge. The interventions were researcher-assigned, and outcome comparisons were based on the content variations. Outcomes were measured using pre/post surveys and electronic medical record data. The study meets the criteria of a randomized controlled trial. | S1. Are there clear research questions? | X |  |  | The study clearly aims to assess the effect of adding quantitative information and/or a nudge affects colorectal cancer screening decisions and behavior. |
|  |  |  | S2. Do the collected data allow to address the research questions? | X |  |  | Data on screening intent, test preference, risk perception, decision conflict, and actual screening uptake were collected and analyzed. |
|  |  |  | 2.1. Is randomization appropriately performed? | X |  |  | Participants were randomly assigned using a computer-generated, stratified allocation and concealed envelopes. |
|  |  |  | 2.2. Are the groups comparable at baseline? | X |  |  | Baseline demographics and outcomes were reported; no significant group differences noted. |
|  |  |  | 2.3. Are there complete outcome data? | X |  |  | Screening uptake and all survey data were reported; missing data were minimal and handled transparently. Statistical testing was performed. Data for comparing the effectiveness between groups were provided. |
|  |  |  | 2.4. Are outcome assessors blinded to the intervention? |  | X |  | The study was open-label; neither participants nor researchers were blinded due to visible differences in the decision aids. |
|  |  |  | 2.5. Did participants adhere to the assigned intervention? | X |  |  | All participants received the assigned module(s), and survey and uptake data were collected as planned. |
| Stoffel et al., 2019 | Quantitative - randomized controlled trial  *Note: Survey experiment* | This study was an online survey experiment in which participants were randomly assigned to one of five message conditions in a hypothetical scenario. The aim was to examine the effects of different formulations of social norm messages—such as verbal quantifiers (“nearly half” vs. “a large number”), numerical values, and a control condition—on screening intentions. The study involved researcher-controlled exposures and statistical comparisons between groups. Conducted under controlled experimental conditions, the study meets the criteria of a randomized controlled trial. | S1. Are there clear research questions? | X |  |  | The study clearly aims to assess how different ways of communicating the current uptake of 43% of the English bowel scope screening program affects intention among disinclined adults. |
|  |  |  | S2. Do the collected data allow to address the research questions? | X |  |  | Data on self-reported intention amd demographics were collected and analyzed. |
|  |  |  | 2.1. Is randomization appropriately performed? | X |  |  | Random allocation was conducted within the survey platform. |
|  |  |  | 2.2. Are the groups comparable at baseline? | X |  |  | Sociodemographics were comparable across groups; any small imbalances were adjusted for in analysis. |
|  |  |  | 2.3. Are there complete outcome data? | X |  |  | Complete screening outcome data were analyzed for all included participants. While some initial participants were excluded based on eligibility or survey quality, no missing outcome data were reported for the final sample. Statistical testing was performed. Data for comparing the effectiveness between groups were provided. |
|  |  |  | 2.4. Are outcome assessors blinded to the intervention? |  | X |  | Outcome data were self-reported by participants, who were aware of their assigned condition; blinding was not feasible. |
|  |  |  | 2.5. Did participants adhere to the assigned intervention? | X |  |  | Participants responded to their assigned message condition. No cross-over or deviations were reported. Message comprehension checks were included. |
| Stoffel et al., 2020 | Quantitative - randomized controlled trial  *Note: Survey experiment* | This study was an online survey experiment in which participants were randomly assigned to either a decoy nudge condition (offering a choice between male and female endoscopists) or a control condition (female-only). The aim was to assess the impact of choice architecture on decision-making. Interventions were researcher-assigned, and outcomes were compared across groups.  Conducted in a hypothetical setting under controlled experimental conditions, the study meets the criteria of a randomized controlled trial. | S1. Are there clear research questions? | X |  |  | The study clearly aims to assess whether offering a male practitioner as a decoy increases women's intentions and choices to undergo colorectal cancer screening with a female endoscopist. |
|  |  |  | S2. Do the collected data allow to address the research questions? | X |  |  | Data on self-reported intentions, discrete choices and demographics were collected and analyzed. |
|  |  |  | 2.1. Is randomization appropriately performed? | X |  |  | Random allocation was conducted within the survey platform. |
|  |  |  | 2.2. Are the groups comparable at baseline? | X |  |  | Sociodemographic characteristics (e.g., age, gender, education) were reported and showed no major differences between groups, indicating baseline comparability. |
|  |  |  | 2.3. Are there complete outcome data? | X |  |  | Outcome data (intention/choice) were reported for all participants who passed the comprehension checks and were randomized. No attrition reported. Statistical testing was performed. Data for comparing the effectiveness between groups were provided. |
|  |  |  | 2.4. Are outcome assessors blinded to the intervention? |  | X |  | Outcome data were self-reported by participants, who were aware of their assigned condition; blinding was not feasible. |
|  |  |  | 2.5. Did participants adhere to the assigned intervention? | X |  |  | Participants responded to the intended scenario(s) and completed the assessments accordingly. No cross-over or deviations were reported. |
| Stoffel et al., 2022 | Quantitative -randomized controlled trial | This population-based trial tested a behavioral intervention comparing standard opt-in letters with enhanced active choice invitations to increase screening participation. Participants were individually randomized using a computer-generated algorithm, and all interventions were researcher-assigned. Screening outcomes such as acceptance and participation were statistically compared between groups.  The study meets the criteria of a randomized controlled trial. | S1. Are there clear research questions? | X |  |  | The study clearly aims to evaluate whether enhanced active choice increases screening acceptance and participation compared to standard opt-in letters. |
|  |  |  | S2. Do the collected data allow to address the research questions? | X |  |  | Data on acceptance (communicating with the screening  program that they want to receive a test kit), participation rates, and demographics were collected and analyzed. |
|  |  |  | 2.1. Is randomization appropriately performed? | X |  |  | Random allocation was performed using a computer-based system. |
|  |  |  | 2.2. Are the groups comparable at baseline? |  |  | X | Limited reporting makes it difficult to assess comparability. Only some baseline characteristics such as age and gender were reported, but no group comparison or statistical testing was presented. Individual-level randomization and sample size suggests likely balance, but reporting is insufficient to confirm. |
|  |  |  | 2.3. Are there complete outcome data? | X |  |  | Outcome data were reported for all included participants; no missing data indicated Statistical testing was performed. Data for comparing the effectiveness between groups were provided. |
|  |  |  | 2.4. Are outcome assessors blinded to the intervention? |  | X |  | Outcomes were self-reported by participants who were aware of their assignment, so blinding was not feasible. |
|  |  |  | 2.5. Did participants adhere to the assigned intervention? | X |  |  | All participants received and responded to their assigned condition; no cross-over or deviations reported. |

1. Enhance individuals to choose between options before progressing to the next stage of the process. Includes active choice, enhanced active choice, forced choice. Based on:

   Keller, P.A., Harlam B.,Loewenstein G., & Volpp K.,G. (2011). Enhanced active choice: a new method to motivate behavior change. *Journal of Consumer Psychology, 21*, 376–383.

   Carroll, G. D., Choi, J. J., Laibson, D., Madrian, B. C., & Metrick, A. (2009). Optimal Defaults and Active Decisions. *The quarterly journal of economics*, *124*(4), 1639–1674. <https://doi.org/10.1162/qjec.2009.124.4.1639>

   Sunstein, C. R. (2017). Forcing people to choose is paternalistic. *Mo. L. Rev.*, *82*, 643. [↑](#footnote-ref-1)
